# Comparison of transcriptomic profiles between HFPO-DA and prototypical PPARα, PPARγ, and cytotoxic agents in mouse, rat, and pooled human hepatocytes

**DOI:** 10.1093/toxsci/kfae044

**Published:** 2024-04-04

**Authors:** Melissa M Heintz, William D Klaren, Alexander W East, Laurie C Haws, Steven R McGreal, Rebecca R Campbell, Chad M Thompson

**Affiliations:** ToxStrategies LLC, Asheville, North Carolina 28801, USA; ToxStrategies LLC, Asheville, North Carolina 28801, USA; ToxStrategies LLC, Asheville, North Carolina 28801, USA; ToxStrategies LLC, Austin, Texas 78731, USA; BioIVT LLC, Kansas City, Kansas 66103, USA; BioIVT LLC, Kansas City, Kansas 66103, USA; ToxStrategies LLC, Katy, Texas 77494, USA

**Keywords:** HFPO-DA (GenX), PFAS, peroxisome proliferator-activated receptor α, (PPARα), mode of action (MOA), hepatocytes, transcriptomics

## Abstract

Like many per- or polyfluorinated alkyl substances (PFAS), toxicity studies with HFPO-DA (ammonium, 2,3,3,3-tetrafluoro-2-(heptafluoropropoxy)-propanoate), a short-chain PFAS used in the manufacture of some types of fluorinated polymers, indicate that the liver is the primary target of toxicity in rodents following oral exposure. Although the current weight of evidence supports the PPARα mode of action (MOA) for liver effects in HFPO-DA-exposed mice, alternate MOAs have also been hypothesized including PPARγ or cytotoxicity. To further evaluate the MOA for HFPO-DA in rodent liver, transcriptomic analyses were conducted on samples from primary mouse, rat, and pooled human hepatocytes treated for 12, 24, or 72 h with various concentrations of HFPO-DA, or agonists of PPARα (GW7647), PPARγ (rosiglitazone), or cytotoxic agents (ie, acetaminophen or d-galactosamine). Concordance analyses of enriched pathways across chemicals within each species demonstrated the greatest concordance between HFPO-DA and PPARα agonist GW7647-treated hepatocytes compared with the other chemicals evaluated. These findings were supported by benchmark concentration modeling and predicted upstream regulator results. In addition, transcriptomic analyses across species demonstrated a greater transcriptomic response in rodent hepatocytes treated with HFPO-DA or agonists of PPARα or PPARγ, indicating rodent hepatocytes are more sensitive to HFPO-DA or PPARα/γ agonist treatment. These results are consistent with previously published transcriptomic analyses and further support that liver effects in HFPO-DA-exposed rodents are mediated through rodent-specific PPARα signaling mechanisms as part of the MOA for PPARα activator-induced rodent hepatocarcinogenesis. Thus, effects observed in mouse liver are not appropriate endpoints for toxicity value development for HFPO-DA in human health risk assessment.

HFPO-DA (ammonium, 2,3,3,3-tetrafluoro-2-(heptafluoropropoxy)-propanoate; CASRN 62037-80-3) is a short-chain polyfluorinated ether used in the manufacture of certain types of fluorinated polymers. HFPO-DA is part of the broader per- and polyfluoroalkyl substances (PFAS) chemical group; although, given the diverse chemical structures of PFAS (eg, functional groups, carbon chain length, and interchain linkages), grouping all PFAS together is likely not appropriate for assessment of human health risk (Anderson *et al.*, 2022). Like many, but not all PFAS, toxicity studies with HFPO-DA indicate that the liver is the primary target of toxicity in rodents following oral exposure. In 2021, the United States Environmental Protection Agency (USEPA) assessed the toxicity of HFPO-DA and developed chronic and subchronic reference doses (RfDs) based on liver lesions in mice from a subchronic oral toxicity study. More recently, the MOA underlying the development of these liver effects in mice was investigated by integrating newly published data not available at the time of USEPA’s assessment with existing data (Heintz *et al.*, 2023). As a part of the assessment by Heintz *et al.* (2023), the updated evidence base for HFPO-DA was assessed in the context of the early Key Events (KEs) outlined in the established MOA framework for peroxisome proliferator-activated receptor alpha (PPARα) activator-induced rodent hepatocarcinogenesis developed by Corton *et al.* (2018). This MOA is under development as an adverse outcome pathway (AOP) (Corton, 2023). Based on this assessment, Heintz *et al.* (2023) concluded that the overall weight of evidence, from mechanistic and phenotypic data, strongly supported that the liver effects in mice following HFPO-DA exposure were occurring via the PPARα MOA and, as such, HFPO-DA-mediated liver changes in mice are likely not relevant for human health risk assessment as it is well-established that only the first KE of the PPARα MOA, PPARα activation, is shared across species (Corton *et al.*, 2018).

Despite the current weight of evidence supporting the PPARα MOA for effects associated with HFPO-DA exposure in mouse liver, alternate MOAs, including those involving cytotoxicity, other PPAR subtypes (eg, PPARγ), and mitochondrial dysfunction, have also been hypothesized based on very limited evidence in the scientific literature (USEPA, 2021). Effects attributed to a MOA involving mitochondrial dysfunction, described as increased mitochondrial number and mitochondrial β-oxidation gene expression in HFPO-DA-exposed mouse livers, are likely PPARα-mediated effects and part of the first KE in the PPARα MOA for rodent liver tumors (see Heintz *et al.*, 2023 for details). Evidence supporting a cytotoxic MOA stems largely from the overinterpretation of single-cell necrosis in H&E-stained liver sections. However, a recent analysis of liver sections demonstrated that hepatocellular death in mouse liver following HFPO-DA exposure could not be clearly discerned in H&E-stained liver sections as cytotoxicity alone. Notably, several examples of cells sharing purported necrotic features stained positive for markers of apoptosis (cleaved caspace-3), indicating these cells were likely apoptotic as opposed to necrotic (Thompson *et al.*, 2023). Moreover, transcriptomic analyses of sections from the same study revealed enrichment for hepatocellular death pathways associated with apoptosis but not cytotoxicity, ferroptosis, pyroptosis, or necroptosis. Evidence for PPARγ-mediated MOA in livers of mice exposed to HFPO-DA is less clear due to crosstalk among PPAR subtypes (eg, α/β/δ/γ) between organs and the overlap in regulation of certain lipid homeostasis-associated genes. Nonetheless, the expression of each PPAR subtype differs by organ, supporting a distinct physiological role for each subtype (Wang *et al.*, 2020).

To further evaluate these MOAs, an *in vitro* transcriptomic study was conducted to compare transcriptomic responses in primary mouse, rat, and human hepatocytes treated with various concentrations of HFPO-DA or agents with known MOAs (ie, positive controls) across several treatment durations. Positive controls included known PPARα and PPARγ agonists (ie, GW7647 and rosiglitazone) (Brown *et al.*, 2001; Lehmann *et al.*, 1995) and known hepatotoxicants (ie, acetaminophen or d-galactosamine). This study design was informed by design elements described in McMullen *et al.* (2020). Specifically, experiments were conducted using pooled human hepatocytes and primary hepatocytes from CD-1 mice, PPARα knockout (KO) mice, B6129SF2/J mice (genetic background strain for PPARα KO mice), and Sprague Dawley rats (Figure 1). Transcriptomic responses in CD-1 mouse hepatocytes are the primary focus of the analyses presented herein and were investigated because this mouse strain was used in various OECD guideline toxicity studies for HFPO-DA. Sprague Dawley rat hepatocytes were used to investigate whether transcriptomic responses to HFPO-DA treatment are consistent across rodent species. PPARα KO mouse hepatocytes and their genetic background strain, B6129SF2/J, were used to further inform the MOA of HFPO-DA. Results presented herein assess the concordance in transcriptomic responses across chemical treatment groups for CD-1 mouse, rat, and human hepatocytes. In addition, the consistency of hepatocyte responses across wild-type (WT) mouse strains, CD-1 and B6129SF2/J, and rats were also evaluated. Transcriptomic analyses comparing treated PPARα KO and WT background strain mouse hepatocytes are presented in a companion publication (see Heintz *et al.*, 2024).

**Figure 1.**
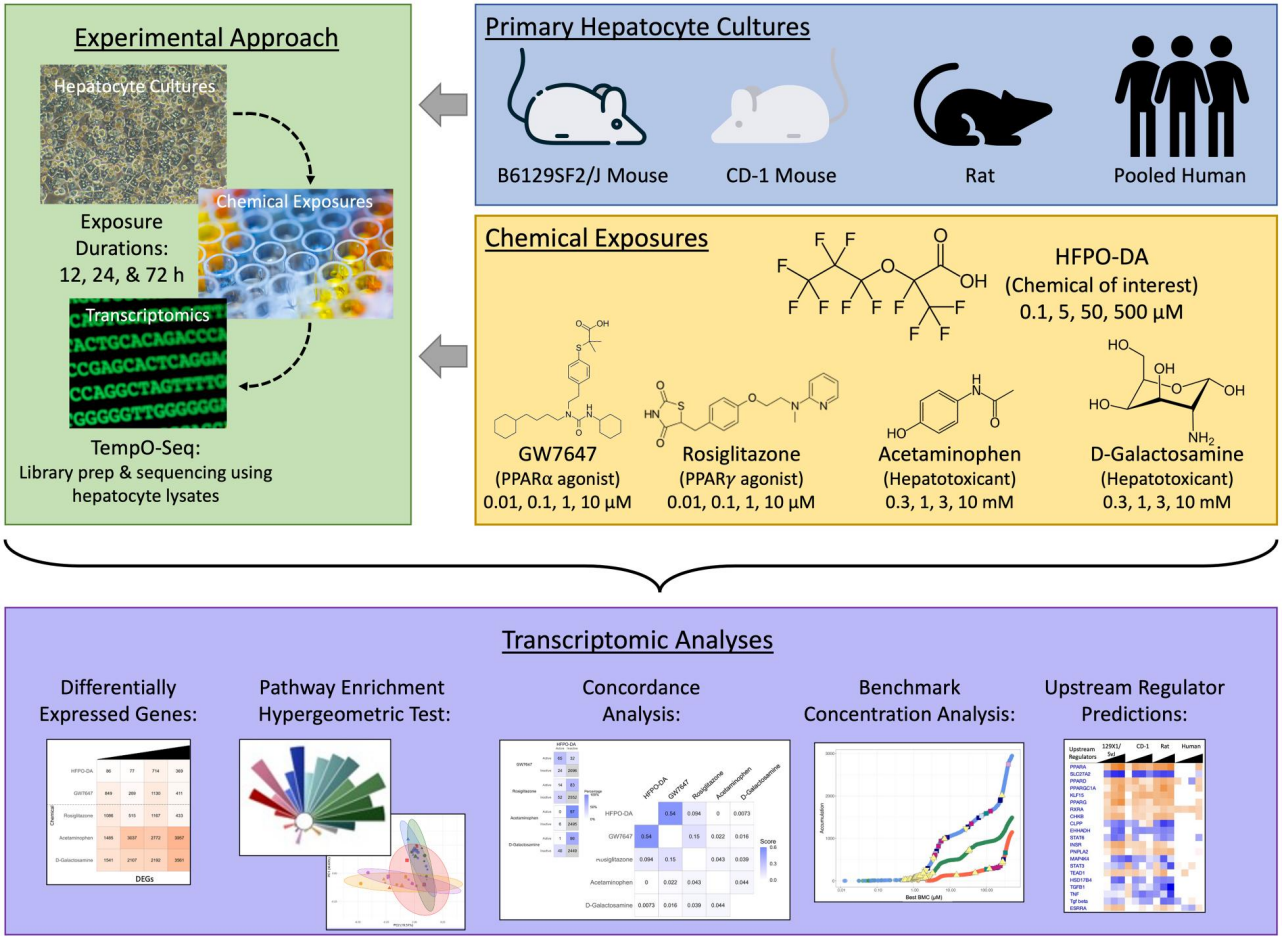
Experimental study design.

## Materials and methods

###  

####  

##### Chemicals

Ammonium perfluoro(2-methyl-3-oxahexanoate) (HFPO-DA; CASRN 62037-80-3; 95% purity) was purchased from Manchester Organics Ltd (Runcorn, Cheshire, UK). GW7647 (CASRN 265129-71-3; ≥98% purity) was purchased from Cayman Chemical Company (Ann Arbor, Michigan). Rosiglitazone (CASRN 122320-73-4; 98.9% purity), acetaminophen (CASRN 103-90-2; ≥99% purity), and d-galactosamine (CASRN 1772-03-8; ≥99% purity) were purchased from Sigma Aldrich (St Louis, Missouri).

##### Primary hepatocyte isolation and culture

Mouse hepatocytes were isolated from the livers of 8–12-week-old male CD-1 mice (CD-1 IGS, strain code: 022) purchased from Charles River (Raleigh, North Carolina) and 11-week-old male B6129SF2/J mice (stock no. 101045) purchased from The Jackson Laboratory (Bar Harbor, Maine). Rat hepatocytes were isolated from the livers of 8–12-week-old male Sprague Dawley (SD, strain code: 001) rats purchased from Charles River. Primary hepatocytes from male mice and rats were used for the *in vitro* assay herein based on findings from previous toxicity studies in rodents demonstrating greater sensitivity to HFPO-DA-mediated liver effects in males (Chappell *et al.*, 2020; Heintz *et al.*, 2022; Thompson *et al.*, 2019). Human hepatocytes were isolated from a pool of 10 donor livers (5 male and 5 female). Additional donor information is available in Supplementary File 1. Hepatocytes from all species/strains were isolated using a 2-step enzymatic digestion of liver tissue as described in Mudra and Parkinson (2001). Hepatocyte viability was determined by trypan blue (0.04%; Millipore Sigma, St Louis, Missouri) exclusion and was ≥79%.

Primary hepatocytes were plated in a collagen-sandwich configuration on 48-well plates. Hepatocytes were maintained in Modified Eagle’s medium, Dr Chee’s modification (MCM; Gibco, Grand Island, New York) supplemented with 25.9 mM NaHCO_3_ (Sigma Aldrich), 953.56 μM l-arginine (Sigma Aldrich), 3.95 mM l-glutamine (Sigma Aldrich), 40.51 μM thymidine (Sigma Aldrich), 0.988% (v/v) ITS+ (6.18 μg/ml insulin, 6.18 μg/ml transferrin, 6.18 ng/ml selenium, 5.29 μg/ml linoleic acid, 1.24 mg/ml BSA; BD Biosciences, Bedford, Massachusetts), 98.8 μg/ml primocin (InvivoGen, San Diego, California), and 0.099 μM dexamethasone (Sigma Aldrich). Cell cultures were incubated in a humidified culture chamber (37 ± 2°C at 95% relative humidity, 95/5% air/CO_2_).

##### Hepatocyte treatments

Using 48-well plates, CD-1 (Experiment was repeated for CD-1 mouse hepatocytes due to a sample mishandling error.) and B6129SF2/J mouse hepatocytes were seeded at a density of 0.6 × 10^6^ cells/ml, and rat and human hepatocytes were seeded at a density of 1.3 × 10^6^ cells/ml. After a 24 h acclimatization period, hepatocytes from each species/strain were treated for 12, 24, or 72 h with supplemented MCM media containing solvent control in the presence or absence of HFPO-DA (0.1, 5, 50, or 500 μM) or one of the following positive controls: GW7647 (0.01, 0.1, 1, or 10 μM), rosiglitazone (0.01, 0.1, 1, or 10 μM), acetaminophen (0.3, 1, 3, or 10 mM) or d-galactosamine (0.3, 1, 3, or 10 mM). Deionized water (1%) served as the solvent control for HFPO-DA and dimethylsulfoxide (DMSO, 0.1%; cell culture grade; Sigma Aldrich) served as the solvent control for the remaining test chemicals. Treatment solutions were replaced every 24 h. For each species/strain, treatment groups were performed in triplicate wells for 12 and 72 h treatment durations, and quadruplicate wells for the 24 h treatment duration.

At 24, 48, and 72 h following treatment, hepatocyte cultures were visualized with a Nikon TMS Microscope (Nikon Corporation) or Accu-Scope 3032 Inverted Microscope (Accu Scope Inc.), and representative hepatocytes from each species/strain treatment group were photographed with a PAXcam5 digital camera (MIS Inc.) to document morphological integrity.

##### Cytotoxicity assay

The release of lactate dehydrogenase (LDH) into the culture medium is an indicator of loss of cell membrane integrity and was used to estimate cytotoxicity in conjunction with visualization of hepatocyte morphological integrity. LDH release was measured using a commercial kit (Roche Diagnostics GmbH, Mannheim, Germany) according to the manufacturer’s directions. Briefly, at 12, 24, and 72 h, medium samples were collected from hepatocytes treated with solvent controls (DMSO or deionized water) only, supplemented MCM only (negative control for LDH assay), or test chemicals. Three untreated hepatocyte samples per species/strain and timepoint were treated with 1% Triton X-100 solution (positive control for LDH assay) and incubated for a period of 30–120 min. Aliquots of each medium sample were transferred to a 96-well plate and mixed with aliquots of the LDH assay working solution to begin the reaction. LDH activity for each medium sample was measured using a spectrophotometer at 490 nm (BioTek Instruments, Inc.). Cytotoxicity in a treatment group was determined based on measurements of percent LDH release ≥25% in combination with changes in hepatocyte morphology indicative of cytotoxicity. A preliminary cytotoxicity assay was performed to select treatment concentrations used in the present study (data not shown).

##### RNA preparation and sequencing

Following treatment with HFPO-DA or positive controls (ie, GW7647, rosiglitazone, acetaminophen or d-galactosamine) for 12, 24, or 72 h, primary hepatocytes for each species/strain were lysed using TempO-Seq Enhanced Lysis Buffer and processed according to the TempO-Seq protocol by BioSpyder Technologies (Carlsbad, California), as described previously (Yeakley *et al.*, 2017). Resultant DNA libraries were sequenced using a HiSeq 2500 Ultra-High-Throughput Sequencing System (Illumina, San Diego, California).

##### Sequencing data processing and assessment of quality

Raw sequencing data (ie, FASTQ files) were analyzed according to the TempO-Seq data analysis pipeline described in Yeakley *et al.* (2017). For each species/strain, the output from the TempO-Seq pipeline was a table containing the number of sequenced reads per TempO-Seq probe per sample, with each probe representing a gene-specific sequence. Samples were excluded from the downstream analyses if either or both of the following exclusion criteria were met: (1) overall sequencing depth (total number of reads across all probes) lower than 2 standard deviations below the mean sequencing depth across all samples from the same species/strain; (2) total number of sequenced probes lower than 2 standard deviations below the mean number of probes sequenced per sample from the same species/strain. Count data from all samples that were not excluded were used for further comparative analyses.

##### Differential gene expression analyses

Sequencing data were analyzed using packages (described here and in subsequent sections) in the R software environment, version 4.3.1 (cran.r-project.org/). Data were normalized using the DESeq2 R package (v1.40.2) (Love *et al.*, 2014) to account for sample-to-sample variation in sequencing depth within each species/strain. Fold-change and differentially expressed probes (DEPs) associated with chemical treatment were determined within DESeq2 by conducting statistical comparisons between treatment groups and controls from the same species/strain and treatment duration. DEPs were defined as those with a false discovery rate (FDR) <10%, based on *p* values adjusted for multiple testing using the Benjamini and Hochberg (BH) procedure (Love *et al.*, 2014); differentially expressed genes (DEGs) were identified from respective DEPs, as some genes (but not all) are represented by multiple probes in the TempO-Seq assay. The expression levels of 21 398 mouse, 20 922 rat, or 19 683 human genes as measured by 30 146 mouse, 22 253 rat, or 22 533 human probes, respectively, were reported from the TempO-Seq assay for each sample.

##### Identification of pathway-level responses to treatment

Biological pathways associated with transcriptomic responses in hepatocytes from each species/strain following treatment with HFPO-DA or positive controls were identified by gene set enrichment analysis. For genes for which multiple probes were used to measure expression, the probe with the highest sequencing count across all samples was used in the pathway analyses. Mouse or rat gene identifiers were converted into human identifiers using the R package biomaRt (v2.56.1) based on the Ensembl genome database (http://uswest.ensembl.org/index.html). Gene expression data were then queried for the enrichment of gene sets within the canonical pathway (CP) subcollection (c2.cp.v2022.1) available through the Molecular Signatures Database (MSigDB; http://software.broadinstitute.org/gsea/msigdb/index.jsp), which includes gene sets from several pathway databases. The enrichment of sets of genes (ie, the constituents of a molecular signaling pathway) was evaluated using the hypergeometric test method for overrepresentation. In comparison with the gene set enrichment analysis (GSEA) method (Subramanian *et al.*, 2005), which ranks all genes without the application of a threshold, the hypergeometric test method employs a more stringent, threshold approach (eg, based on DEG significance) for inclusion of genes into the gene set enrichment analysis. Due to the complex nature (eg, several species/strains, chemicals, concentrations, and timepoints) of this large *in vitro* study, the application of the hypergeometric test method to the large differential gene expression datasets generated by DESeq2 herein helped to streamline data processing, interpretation, and comparison between test chemicals. DEGs (ie, genes with an FDR of <10% as described above) for each treatment group within a species/strain and timepoint were tested for overrepresentation among the gene sets in the CP subcollection using the Fisher combined probability test function within the Platform for Integrative Analysis of Omics data (PIANO) R package (v2.16.0) (Varemo *et al.*, 2013). Gene sets with an FDR <5% were considered significantly enriched.

##### Gene set concordance Analysis

To understand the degree of similarity or consistency in significantly enriched gene sets between HFPO-DA and positive control-treated hepatocytes at each timepoint for each species/strain, activity concurrence matrices and subsequent concordance analyses were conducted. As chemical treatments occurred at different concentrations for each chemical tested, labels of “low,” “medium,” “medium-high,” and “high” were assigned to the 4 concentration groups for each chemical (eg, for HFPO-DA: 0.1 μM = low, 5 μM = medium, 50 μM = medium-high, 500 μM = high). The medium and medium-high treatment groups for each chemical were selected to use in the main gene set activity concurrence and concordance analyses to ensure that a maximum transcriptomic response was evaluated but avoided concentrations where cytotoxicity was more frequently observed, that is, in the high concentration groups for some timepoints and some chemicals. In order to ensure that concordance results were not affected by cytotoxicity observed at the medium-high concentration (ie, 3 mM) in acetaminophen and d-galactosamine-treated hepatocytes at later timepoints, gene set activity concurrence and concordance analyses were also performed between HFPO-DA, GW7647 or rosiglitazone at the medium-high treatment concentration for each timepoint, and acetaminophen or d-galactosamine at the medium treatment concentration (ie, 1 mM) for each timepoint (see Supplementary Materials).

Using the gene set enrichment results calculated using the hypergeometric test method, upregulated gene sets from the medium-high (or medium) treatment group for each chemical at each timepoint were first filtered for gene sets that contained at least 1 significant DEG (FDR <10%). Next, filtered lists of upregulated gene sets for each chemical/timepoint were converted to binary format by giving gene sets that were significantly enriched (FDR <5%) a value of 1, and gene sets that were not significantly enriched (FDR > 5%) a value of 0. Gene sets with a value of 1 were considered “active,” and gene sets with a value of 0 were considered “inactive.” Activity concurrence matrices were generated from these binary gene set lists according to Table 1.

**Table 1.** Gene set activity concurrence matrix comparing number of active/inactive gene sets in Chemical A to Chemical B

		Chemical A	
		Active	Inactive
**Chemical B**	**Active**	Number of the same gene sets active for both Chemical A and Chemical B	Number of gene sets active for Chemical B but not for Chemical A
	**Inactive**	Number of gene sets active for Chemical A but not for Chemical B	Number of gene sets inactive for Chemical A and Chemical B

Note: The percentage of active gene sets in each matrix quadrant out of the total number of active gene sets between both chemicals in the comparison was also calculated.

Gene set activity concurrence results were then used to calculate concordance indices for each chemical comparison using the Jaccard index method, which has been previously evaluated and applied to transcriptomic data (Black *et al.*, 2022; Shah *et al.*, 2022). The Jaccard index (see equation 1) does not consider the number of gene sets where inactivity was determined for both chemicals, and therefore provides a clearer picture of overlapping active transcriptomic responses at the pathway level.

(1) $$\mathrm{Jaccard}\;\mathrm{Index}=\frac{{\mathrm{Active}}_{A\&B}}{\left(\left({\mathrm{Active}}_{A\&B}\right)+\left({\mathrm{Active}}_{A}|{\mathrm{Inactive}}_{B}\right)+\left({\mathrm{Inactive}}_{A}|{\mathrm{Active}}_{B}\right)\right)}$$

The higher the Jaccard index (ie, closer to 1), the more similar the transcriptomic responses are between 2 chemicals, where 1 indicates complete similarity (identical sets), and 0 indicates no similarity (completely disjoint sets). The same methodology was repeated to assess gene set activity concurrence and concordance for downregulated gene sets.

##### Gene set aggregation and visualizations

To better understand the types of gene sets that were significantly enriched across chemical treatment groups, a comparative targeted gene set analysis was conducted. The top 10 chemical-gene interactions from the Comparative Toxicogenomics Database (CTD; https://ctdbase.org/; accessed November 2022) for HFPO-DA and each of the positive control chemicals were used to identify and select gene sets that contained 1 or more of these top interacting genes (eg, gene sets that contain ACOX1) (see Supplementary Material). CTD is a database curated from data (including transcriptomics data) available in the published literature; the top chemical-gene interactions curated by CTD for each chemical provide insight into the types of mechanisms by which each chemical exerts its effects. From the list of gene sets containing 1 or more interacting genes, adjusted *p* values for significantly enriched gene sets (FDR <5%) across all treatment groups were reversed log-scaled to equal a value greater than 0 and less than or equal to 1, with non-significant pathways set equal to zero, and the gene set with the lowest adjusted *p* value (ie, most significant) set equal to 1. Within each treatment group, gene sets containing the same interacting gene were aggregated by summing the reverse log-scaled adjusted *p* values (eg, all reverse log-scaled adjusted *p*-values for gene sets containing ACOX1 were summed for HFPO-DA, at 0.1 μM, at 24 h in CD-1 mouse hepatocytes). Lastly, the summed totals for each interacting gene for each treatment group and timepoint were scaled from 0 to 1 using both internal and external scaling methods. Internal scaling was defined as scaling the summed totals for each interacting gene relative to each other within the same treatment group and timepoint. External scaling was defined as scaling the summed totals for the same interacting gene across all treatment groups and timepoints within a species/strain. Internal scaling allows for an understanding of the most significantly enriched gene sets within an experimental treatment group, giving insight into potential underlying mechanisms. Whereas external scaling across all treatment groups within a species/strain allows for the investigation of how experimental groups within a species/strain compare with each other for a specific gene. Once data were appropriately scaled, ToxPi visualizations were generated using ToxPi software (https://toxpi.org/; v2.3).

##### Upstream regulator prediction Analysis

Ingenuity Pathway Analysis (IPA, v. 01-22-01; Qiagen Bioinformatics, Redwood City, California) was used to identify predicted upstream regulators associated with DEGs for each treatment group within a species/strain and timepoint. Fold change and statistical values determined by DESeq2 were used to conduct the analyses, specifically DEGs with FDR < 10%.

##### Benchmark concentration Analysis

Concentration-response modeling was conducted using the BMDExpress software (v2.3) (Phillips *et al.*, 2019). Normalized expression data for all samples, as generated using DESeq2, were loaded into BMDExpress without transformation, using probe IDs from the TempO-Seq experiment as gene identifiers. A Williams trend test (with *p* value cutoff = .05) was used to identify genes altered by chemical treatment for each species/strain and timepoint. No fold-change filters or corrections for multiple tests were applied. Benchmark concentration (BMC) analysis was conducted using the following models: linear, power, hill, 2° and 3° polynomial, and exponential models 2–5. The models were run assuming constant variance and a benchmark response (BMR) of 1 standard deviation. Concentration-responsive genes with a best BMC >10-fold below the lowest concentration or a best BMC > the highest concentration were removed (NTP, 2018). Functional classification was conducted using the Reactome gene set collections available within the BMDExpress software, based on significantly concentration-responsive genes (ie, genes with a winning model fit *p* value ≥.1), and removing genes according to the default parameters as follows: genes with BMC/BMCL >20, BMCU/BMC >20, and BMCU/BMCL >40 (NTP, 2018). No filters for minimum or maximum number of genes per gene set were applied. Benchmark concentrations for the gene sets were also calculated.

## Results

###  

#### Transcriptomic changes across species/strains associated with treatment

Following assessment of sequencing data quality using the criteria described in the Materials and methods section, 5 samples each were removed from the analysis for CD-1 mouse, rat, and human hepatocytes, and 7 samples were removed for B6129SF2/J mouse hepatocytes due to low sequencing quality; in total, 22 out of 840 samples were removed across all species/strains. Final sample numbers for each treatment group included in downstream transcriptomic analyses are provided in Supplementary Table 1 and File 1. In general, samples removed across species/strains were from different chemical treatment groups and timepoints; however, at 72 h, all 3 10 mM (highest concentration tested) samples for acetaminophen in CD-1 and B6129SF2/J mouse hepatocytes and d-galactosamine in rat hepatocytes did not meet sequencing data quality criteria and were removed from the analysis. Based on cytotoxicity results (available in Supplementary File 2 and Figure 1), it is not surprising that these samples for acetaminophen and d-galactosamine failed to meet sequencing quality criteria in mouse and rat hepatocytes, respectively. In addition, at 12 h, 2 of the 3 total samples from the 10 mM acetaminophen treatment group in human hepatocytes did not meet sequencing data quality criteria, as a result, this treatment group was also removed from the analysis. Cytotoxicity was primarily observed in hepatocytes treated with acetaminophen or d-galactosamine, the positive controls for a cytotoxic MOA. As expected, cytotoxicity increased with exposure duration (ie, later timepoints, 24 and 72 h) and concentration (ie, 3 and 10 mM) for acetaminophen and d-galactosamine across hepatocyte species/strains (Supplementary File 2 and Figure 1).

**Figure 2.**
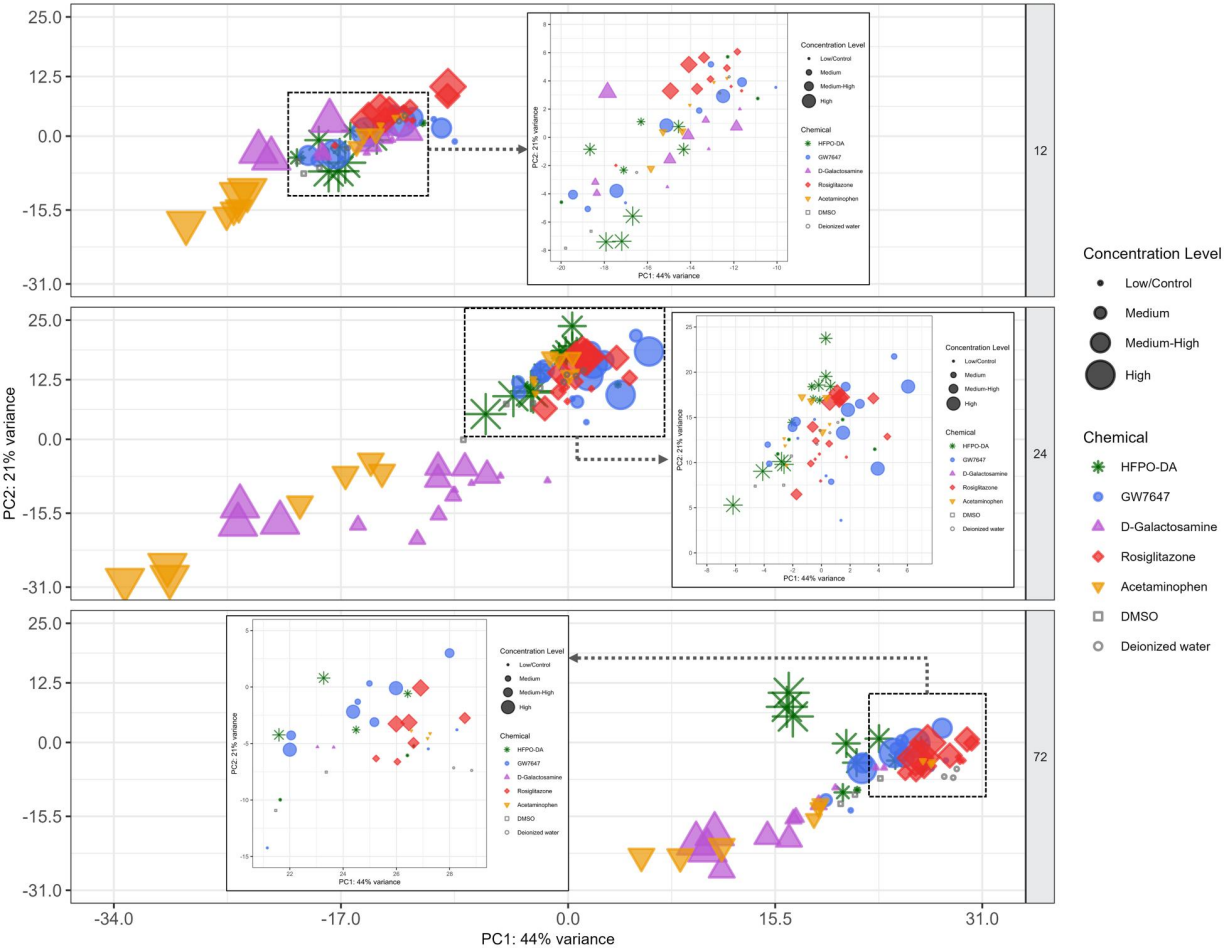
Principal component analysis plot of CD-1 mouse hepatocyte samples. The chemical treatment group of each sample is indicated by color-coded shapes, with shape and color indicating chemical treatment and size indicating concentration level (see legend). When a tighter cluster of samples was observed within a timepoint, secondary PCA plots were generated to further investigate sample clustering patterns. Results for B6129F2/J mouse, rat, and human hepatocytes are available in Supplementary Figure 1.

**Figure 3.**
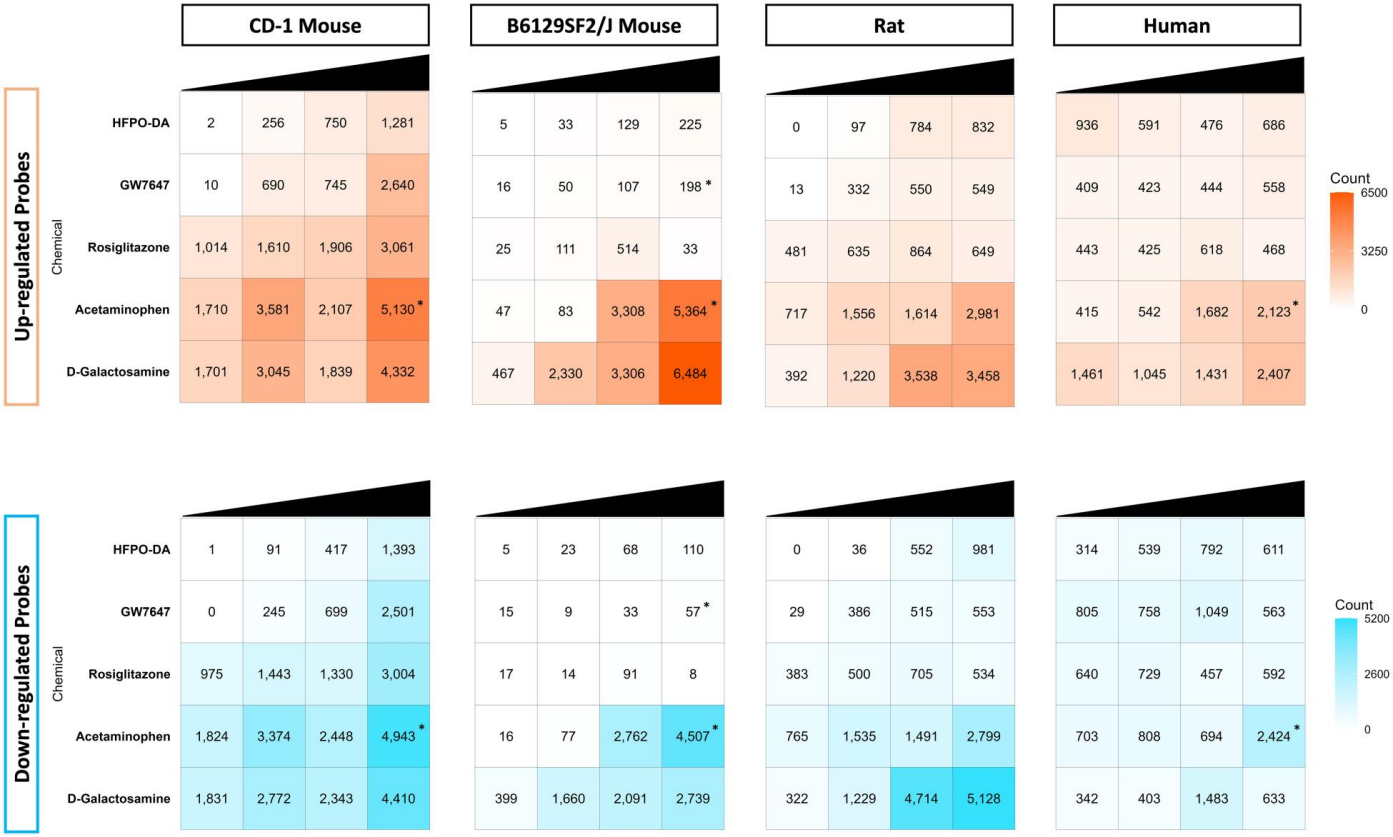
The number of significant (FDR < 10%) upregulated (top row) and downregulated (bottom row) DEPs (relative to controls) in primary hepatocytes for each species/strain and chemical tested at 24 h. Each row represents a different chemical, and each column represents a different test concentration, with concentrations increasing from left to right. An “*” indicates that cytotoxicity was observed at this concentration and timepoint. Results for 12 and 72 h timepoints are available in Supplementary Figure 2.

The variance in transcriptomic profiles between each sample from a species/strain across chemical treatment groups and timepoints was visualized using principal component analysis (PCA) (Figure 2 and Supplementary Figure 2). Within each species/strain, treatment duration (ie, timepoint) contributed the greatest variance between samples, regardless of chemical treatment. Apart from timepoint, variation was also observed between samples according to their chemical treatment group, with samples treated with cytotoxic agents acetaminophen and d-galactosamine having the greatest separation from the other chemical groups. This pattern was consistent in PCA plots containing samples from all timepoints together as well as PCA plots separated by timepoint (Supplementary Figs. 2 and 3). Samples from HFPO-DA, GW7647, and rosiglitazone treatment groups generally clustered more closely with solvent control samples within each timepoint. PCA results were consistent with hierarchical clustering patterns observed within each species/strain (Supplementary Figs. 4A–D); in addition, as expected, samples grouped by species followed by timepoint when all samples were analyzed together by hierarchical clustering (Supplementary Figure 4E). A more distinct separation between chemical treatment groups was observed at the 24 h timepoint as shown in Figure 2, indicating a greater difference between transcriptomic responses across treatment groups at 24 h. For this reason, subsequent analyses focused primarily on the results at 24 h. The number of significant differentially expressed probes (DEPs) between chemical treatment groups at 24 h across species/strains are presented in Figure 3, with results for 12 and 72 h provided in Supplementary Figure 5 and File 3. Compared with other chemicals, acetaminophen, and d-galactosamine generally had the greatest number of DEPs and differentially expressed genes (DEGs) at each timepoint.

Overall, treatment duration had the greatest impact on variance in transcriptomic profiles between hepatocyte samples. Compared with the other chemicals tested, hepatocytes treated with acetaminophen or d-galactosamine generally had a different and greater transcriptomic response based on PCA and differential gene expression results.

#### Comparison of pathway-level responses to HFPO-DA and positive controls in rodent hepatocytes

Gene set enrichment analysis results using the hypergeometric test method are available in Table 2 for CD-1 mouse hepatocytes and Supplementary File 4 for other species/strains. Results for the top significantly enriched gene sets in primary mouse and rat hepatocytes treated with HFPO-DA were similar to results from *in vivo* studies investigating transcriptomic responses to HFPO-DA exposure in mouse liver (Chappell *et al.*, 2020; Heintz *et al.*, 2022), with gene sets such as “KEGG Fatty Acid Metabolism,” “KEGG PPAR Signaling Pathway,” “KEGG Peroxisome,” and “WP Fatty Acid Beta-oxidation” among the top most significantly enriched gene sets across treatment concentrations and timepoints for HFPO-DA. Although downregulated gene sets for HFPO-DA-treated rodent hepatocytes had lower enrichment based on adjusted *p* value compared with upregulated gene sets, downregulation of gene sets related to innate immune responses including cytokine signaling and complement and coagulation cascade (consistent with the aforementioned *in vivo* studies), as well as gene sets related to cell cycle regulation and apoptosis was observed. For GW7647-treated rodent hepatocytes, the most significantly enriched gene sets were essentially identical to that of the top significantly enriched gene sets for HFPO-DA (see Table 2). A lower number of downregulated gene sets were significantly enriched for GW7647 compared with HFPO-DA-treated rodent hepatocytes, however, these significant downregulated gene sets were similar between chemistries. Interestingly, PPARγ-specific gene sets were not significantly enriched in rosiglitazone-treated hepatocytes in any species investigated. These findings are consistent with the low expression of PPARγ reported in the liver (Chawla *et al.*, 1994). Although gene set enrichment patterns were inconsistent across timepoints and treatment groups for rosiglitazone-treated rodent hepatocytes, metabolism of fatty acids and amino acids, as well as regulation of cell proliferation were typically among the most significantly enriched gene sets.

**Table 2.** Top 5 most significantly enriched gene sets determined by the hypergeometric test method at 24 h in CD-1 mouse hepatocytes

Chemical	Concentration	Gene set name	Adjusted *p* value	Direction
HFPO-DA	0.1 µM	REACTOME Fatty Acid Metabolism	0.005342207	Up
		WP Omega-3/Omega-6 Fatty Acid Synthesis	0.005342207	Up
		KEGG Biosynthesis of Unsaturated Fatty Acids	0.007832497	Up
		WP 7-oxo-C and 7-beta-HC Pathways	0.008923765	Up
		REACTOME Mitochondrial Fatty Acid Beta-oxidation	0.013539047	Up
	5 µM	KEGG Fatty Acid Metabolism	3.76E-23	Up
		REACTOME Fatty Acid Metabolism	4.99E-21	Up
		KEGG PPAR Signaling Pathway	1.84E-15	Up
		WP PPAR Signaling Pathway	2.13E-14	Up
		KEGG Peroxisome	2.77E-13	Up
	50 µM	REACTOME Fatty Acid Metabolism	1.66E-22	Up
		KEGG Fatty Acid Metabolism	1.58E-18	Up
		KEGG PPAR Signaling Pathway	2.40E-17	Up
		KEGG Peroxisome	5.57E-17	Up
		WP Fatty Acid Beta-oxidation	1.23E-15	Up
	500 µM	REACTOME Fatty Acid Metabolism	1.43E-17	Up
		KEGG Fatty Acid Metabolism	5.85E-13	Up
		WP Fatty Acid Beta-oxidation	1.53E-10	Up
		KEGG Peroxisome	3.95E-10	Up
		REACTOME Peroxisomal Lipid Metabolism	9.00E-10	Up
GW7647	0.01 µM	KEGG Fatty Acid Metabolism	0.01419192	Up
	0.1 µM	REACTOME Fatty Acid Metabolism	1.27E-23	Up
		KEGG Fatty Acid Metabolism	2.61E-17	Up
		KEGG PPAR Signaling Pathway	2.61E-17	Up
		WP PPAR Signaling Pathway	1.56E-15	Up
		KEGG Peroxisome	9.89E-13	Up
	1 µM	REACTOME Fatty Acid Metabolism	6.37E-26	Up
		KEGG Fatty Acid Metabolism	5.58E-17	Up
		KEGG_PPAR Signaling Pathway	5.58E-17	Up
		WP Fatty Acid Beta-oxidation	9.03E-17	Up
		WP PPAR Signaling Pathway	2.89E-15	Up
	10 µM	REACTOME Fatty Acid Metabolism	2.39E-14	Up
		REACTOME Regulation of Expression of SLITs and ROBOs	7.11E-08	Up
		KEGG PPAR Signaling Pathway	1.97E-07	Up
		REACTOME Regulation of PTEN Stability and Activity	1.97E-07	Up
		REACTOME Signaling by ROBO Receptors	2.16E-07	Up
Rosiglitazone	0.01 µM	REACTOME Nuclear Events Mediated by NFE2L2	0.00078315	Up
		REACTOME KEAP1 NFE2L2 Pathway	0.00114571	Up
		REACTOME Regulation of mRNA Stability by Proteins that Bind AU-Rich Elements	0.00119558	Up
		WP Oncostatin M Signaling Pathway	0.00163657	Up
		BIOCARTA EGF Pathway	0.00296221	Up
	0.1 µM	REACTOME Apoptosis	0.00232271	Up
		REACTOME Nuclear Events Mediated by NFE2L2	0.00232271	Up
		REACTOME Programmed Cell Death	0.00232271	Up
		REACTOME KEAP1 NFE2L2 Pathway	0.00232469	Up
		REACTOME Regulation of PTEN Stability and Activity	0.00232469	Up
	1 µM	WP Amino Acid Metabolism	0.00070216	Up
		BIOCARTA EGF Pathway	0.00405762	Up
		BIOCARTA PDGF Pathway	0.00405762	Up
		KEGG Drug Metabolism Other Enzymes	0.00405762	Up
		PID HIF1 TF pathway	0.00405762	Up
	10 µM	REACTOME Apoptosis	0.00058715	Up
		REACTOME Cellular Response to Chemical Stress	0.00108248	Up
		REACTOME Nuclear Events Mediated by NFE2L2	0.00108248	Up
		REACTOME Programmed Cell Death	0.00108248	Up
Acetaminophen	0.3 mM	REACTOME Cell Cycle Checkpoints	3.06E-05	Up
		REACTOME Nuclear Events Mediated by NFE2L2	3.06E-05	Up
		REACTOME APC/C Mediated Degradation of Cell Cycle Proteins	5.21E-05	Up
		REACTOME_Cyclin A: CDK2 Associated Events at S Phase Entry	0.00022031	Up
		REACTOME KEAP1 NFE2L2 Pathway	0.00022031	Up
	1 mM	REACTOME Nuclear Events Mediated by NFE2L2	2.75E-05	Up
		REACTOME APC/C Mediated Degradation of Cell Cycle Proteins	5.93E-05	Up
		REACTOME Cellular Response to Chemical Stress	5.93E-05	Up
		REACTOME KEAP1 NFE2L2 Pathway	5.93E-05	Up
		PID FOXO Pathway	6.56E-05	Up
	3 mM	KEGG Complement & Coagulation Cascades	2.38E-06	Down
		REACTOME Activation of Gene Expression by SREBF/SREBP	2.38E-06	Down
		WP VEGF/VEGFR2 Signaling Pathway	1.23E-05	Down
		WP Cholesterol Biosynthesis Pathway	4.37E-05	Down
		REACTOME Recycling of Bile Acids & Salts	4.46E-05	Down
	10 mM	REACTOME Response of EIF2AK4 (GCN2) to Amino Acid Deficiency	6.35E-07	Up
		REACTOME Cellular Response to Starvation	6.48E-07	Up
		REACTOME Eukaryotic Translation Initiation	4.41E-06	Up
		REACTOME Nonsense Mediated Decay (NMD)	1.93E-05	Up
		REACTOME Eukaryotic Translation Elongation	2.01E-05	Up
d-Galactosamine	0.3 mM	REACTOME rRNA Processing	5.83E-09	Up
		REACTOME rRNA Modification in the Nucleus and Cytosol	1.11E-07	Up
		WP Photodynamic Therapy-induced Unfolded Protein Response	0.00012449	Up
		REACTOME tRNA Modification in the Nucleus and Cytosol	0.00027087	Up
		REACTOME RMTS Methylate Histone Arginines	0.00050058	Up
	1 mM	REACTOME Metabolism of Amino Acids and Derivatives	2.83E-10	Down
		KEGG Complement & Coagulation Cascades	3.47E-08	Down
		KEGG Peroxisome	2.36E-07	Down
		REACTOME Plasma Lipoprotein Remodeling	4.47E-07	Down
		WP Amino Acid Metabolism	9.16E-07	Down
	3 mM	REACTOME rRNA Modification in the Nucleus & Cytosol	9.98E-11	Up
		REACTOME rRNA Processing	1.57E-10	Up
		REACTOME Translation	1.13E-08	Up
		REACTOME Mitochondrial Translation	1.27E-07	Up
		WP Photodynamic Therapy-induced Unfolded Protein Response	0.00089881	Up
	10 mM	REACTOME rRNA Processing	4.31E-14	Up
		KEGG Oxidative Phosphorylation	7.49E-12	Up
		WP Electron Transport Chain OXPHOS System in Mitochondria	2.91E-10	Up
		REACTOME Respiratory Electron Transport, ATP Synthesis by Chemiosmotic Coupling, and Heat Production by Uncoupling Proteins	3.53E-10	Up
		REACTOME Respiratory Electron Transport	3.22E-09	Up

For acetaminophen and d-galactosamine, the positive controls for liver cytotoxicity, the top significantly enriched gene sets included those related to RNA processing and translation and regulation of the cell function (eg, Slit/Robo signaling). In general, as demonstrated in Table 2, adjusted *p* values for the most significantly enriched gene sets for HFPO-DA and GW7647-treated rodent hepatocytes were substantially lower (ie, several orders of magnitude lower) than the adjusted *p* values for the most significantly enriched gene sets in rodent hepatocytes treated with rosiglitazone or cytotoxic positive controls.

Compared with the number of significantly enriched gene sets in primary mouse and rat hepatocytes, few to no gene sets were enriched consistently across all chemical treatment groups and timepoints in primary human hepatocytes. As such, gene set concordance between HFPO-DA and positive controls could not be evaluated in human hepatocytes. As demonstrated below, analyses in human hepatocytes were therefore mostly limited to individual genes.

**Figure 4.**
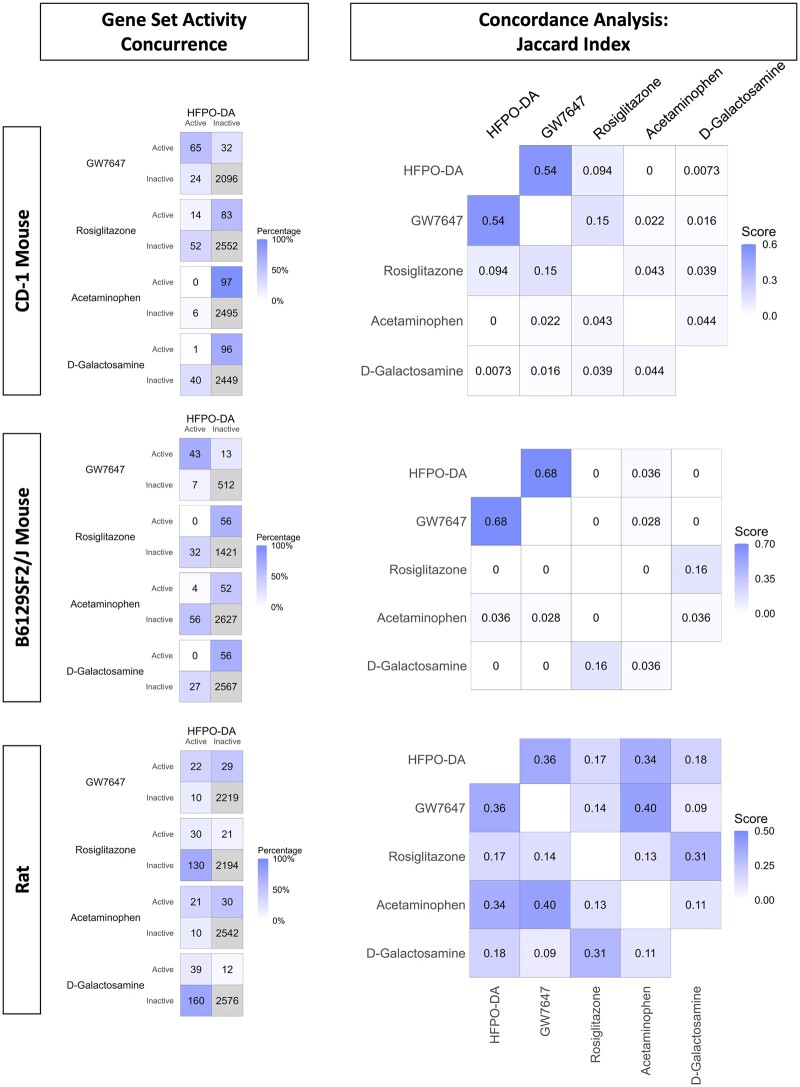
Upregulated gene set activity concurrence and concordance analyses at 24 h. Comparison of gene set activity concurrence and concordance results across CD-1, B6129SF2/J and rat hepatocytes at the second-highest concentration tested for each chemical at 24 h. Activity concurrence matrices show the number of instances when there is agreement and disagreement in the active/inactive (ie, significant/not significant) upregulated gene sets, comparing HFPO-DA to each positive control. The number within each quadrant of the concurrence matrices provides the number of active/inactive gene sets for both chemicals, whereas the purple shading within the concurrence matrices indicates the percentage of active gene sets in a particular quadrant out of the total number of active gene sets between both chemicals in the comparison. The number of gene sets inactive for both chemicals (inactive-inactive) are shaded gray (lower right quadrant) and were not included in the percentage calculation. The calculation of the percent of active gene sets allows for a more appropriate comparison across the positive controls for active gene sets in common (ie, gene sets active for both chemicals) or different (ie, gene sets active for 1 chemical and inactive for the other) with HFPO-DA. The Jaccard index method was then applied to activity concurrence results to estimate relative concordance of active gene sets between HFPO-DA and each positive control. Jaccard indices comparing positive controls to each other are also provided for context. Greater color intensity and higher scores for concordance plots indicate greater overlap between active gene sets for 2 chemicals.

To examine the degree of overlap and agreement in gene set enrichment between HFPO-DA and positive controls for each rodent species/strain, activity concurrence matrices were generated and, subsequently, concordance calculated using the Jaccard index. This was done for upregulated gene sets at the medium and medium-high concentrations tested. Additional gene set activity concurrence and concordance analyses were also performed between HFPO-DA, GW7647, or rosiglitazone at the medium-high treatment concentration for each timepoint, and acetaminophen or d-galactosamine at the medium treatment concentration (ie, 1 mM) for each timepoint (see Materials and methods). Given the small number of downregulated gene sets in rodent hepatocytes treated with GW7647 and rosiglitazone (Supplementary File 4) these analyses focused only on upregulated gene sets. As expected with large transcriptomic datasets, most of the gene set agreements between HFPO-DA and each positive control in activity concurrence analyses were driven by gene sets that were inactive (ie, non-significantly enriched) for both chemicals. However, to estimate gene set concordance, the Jaccard index (see Materials and methods) does not consider inactive gene sets in both chemistries but focuses on the active (ie, significantly enriched) gene sets for each chemical. The highest concordance scores for active upregulated gene sets in both mouse and rat primary hepatocytes were consistently between HFPO-DA and GW7647 across all 3 timepoints and treatment concentrations evaluated—indicating a substantial degree of similarity in the transcriptomic profiles for HFPO-DA and GW7647 (see Figure 4 for medium-high concentration at 24 h timepoint; see Supplementary Figure 6 for other timepoints and concentration comparisons; data not shown for medium-only concentration comparisons).

**Figure 5.**
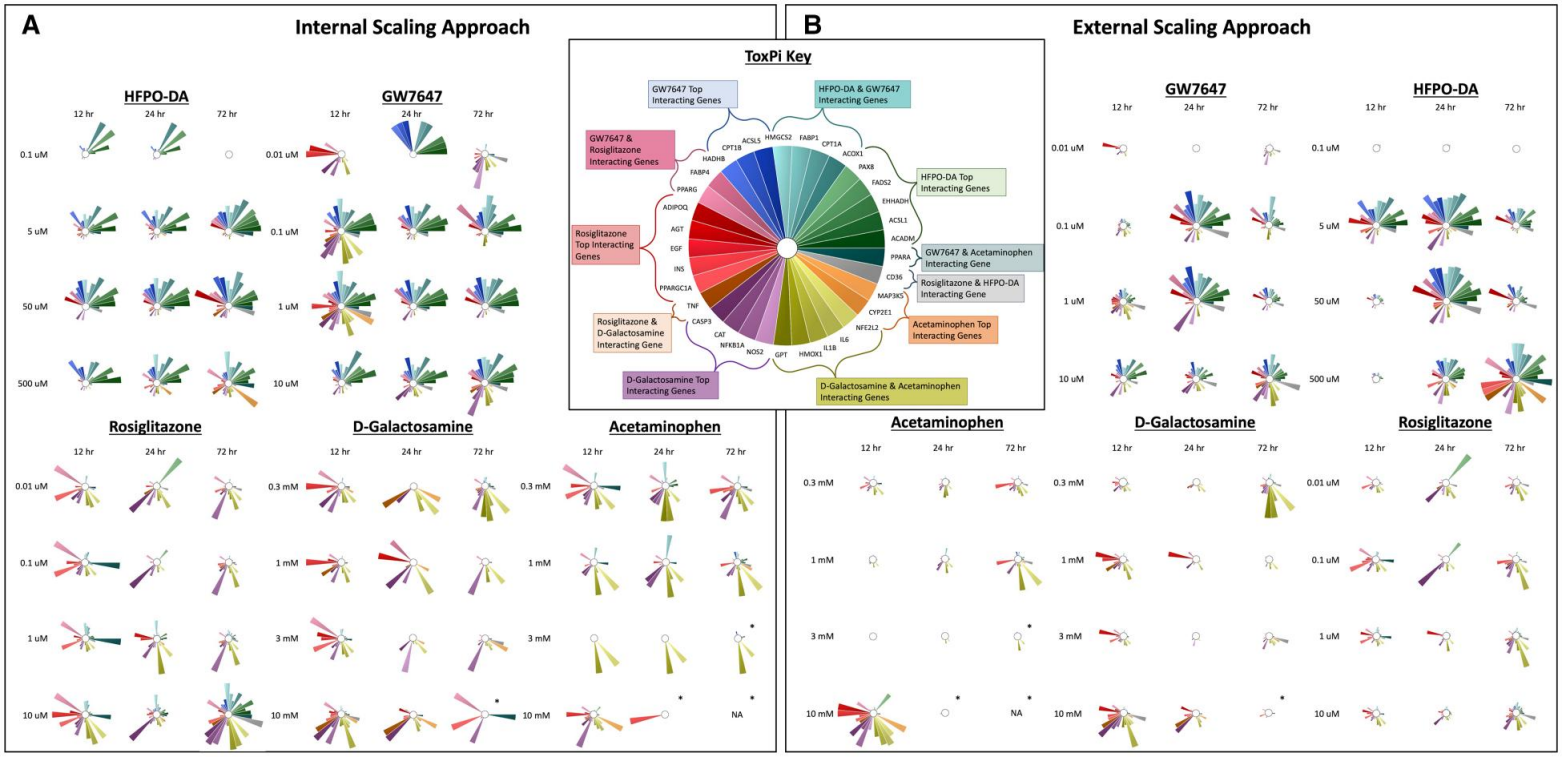
ToxPi visualizations of upregulated gene set aggregation results for CD-1 mouse hepatocytes. Significant (FDR < 5%) upregulated gene sets from hypergeometric gene set enrichment analysis containing genes known to interact with HFPO-DA and/or positive controls were aggregated as described in the Materials and methods section. The size of a ToxPi wedge for a given gene reflects the significance and number of enriched gene sets containing that gene within a specific chemical treatment group and timepoint that is (A) scaled in respect to the other wedges for different genes within the same ToxPi (ie, internal scaling) or is (B) scaled in respect to the same gene wedge across ToxPis for different chemical treatment groups/timepoints (ie, external scaling). An “*” indicates that cytotoxicity was observed at this concentration and timepoint. An empty ToxPi indicates that none of the targeted gene sets were enriched significantly, and “NA” means that samples from this concentration and timepoint did not undergo transcriptomic analyses due to low sequencing quality from extensive cytotoxicity.

**Figure 6.**
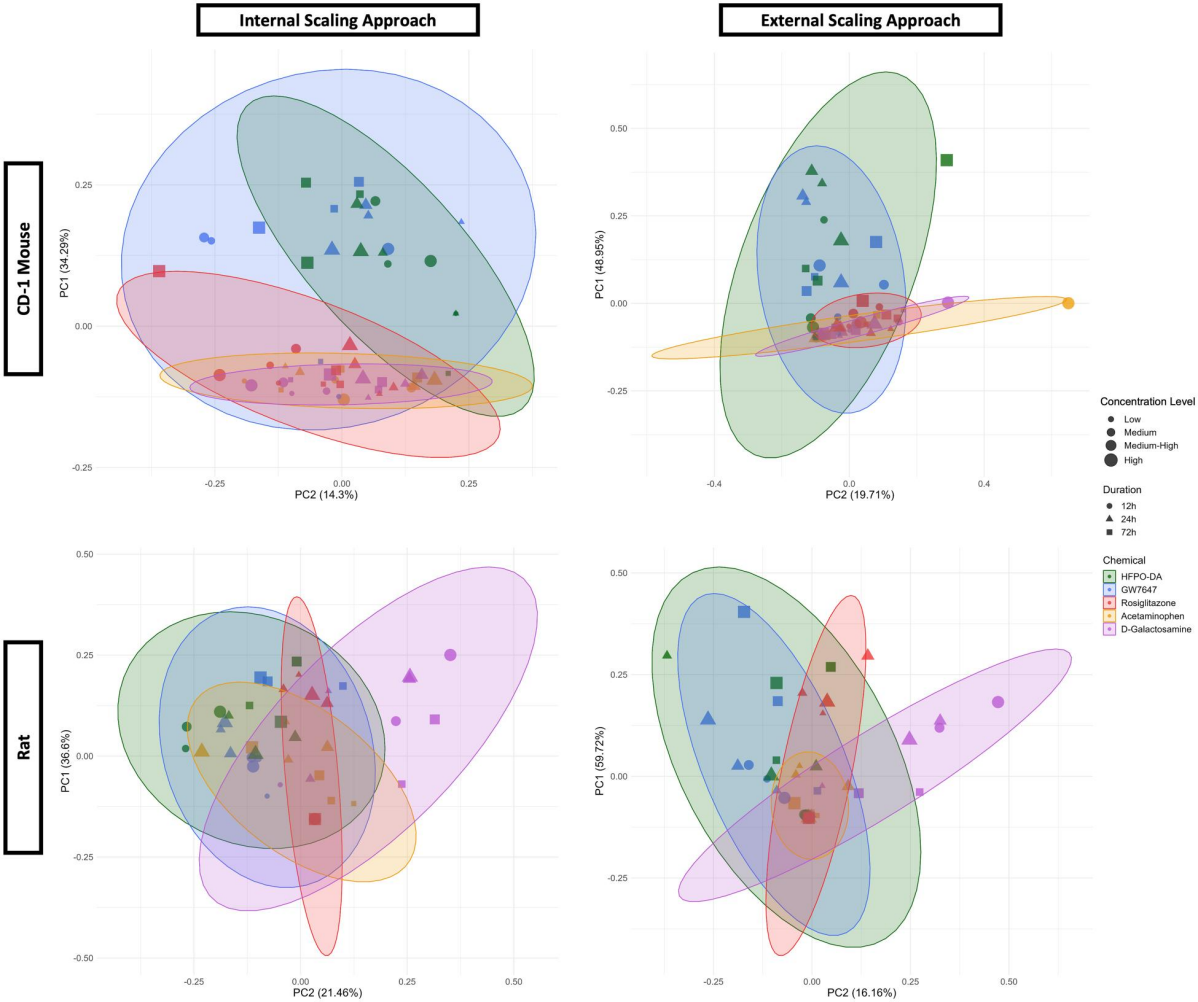
PCA of upregulated gene set aggregation results for CD-1 mouse and rat hepatocytes using internal and external scaling methods. Shaded ellipses are based on the covariance matrix of the aggregated gene set results with ellipse size accounting for aspects of the underlying cumulative probability distribution, ie the ellipses provide general insight into the directionality and variance of the respective chemical treatment groups. Results for B6129SF2/J mouse hepatocytes available in companion publication.

Assessment of concordance between HFPO-DA and other positive controls at the pathway level in rodent hepatocytes was further analyzed by gene set aggregation of known chemical-gene interactions. Gene set aggregation visuals (ie, ToxPi visuals, PCA plots) for CD-1 mouse hepatocytes demonstrated homogeneous upregulated gene set enrichment profiles for HFPO-DA and GW7647 (ie, similar ToxPi wedge pattern and size across treatment concentrations and timepoints), with a stark difference in comparison with rosiglitazone (PPARγ agonist) and the cytotoxic agents (acetaminophen and d-galactosamine). This conclusion is based on both internal (within a specific chemical treatment group and timepoint for a species/strain) and external (across chemical treatment groups and timepoints for a species/strain) scaling approaches as described in the Materials and methods section (Figure 5). PCA plots of the gene set aggregations using both internal and external scaling methods for mouse (CD-1) and rat hepatocytes demonstrated that HFPO-DA and GW7647 clusters generally had the greatest overlap between each other, confirming the ToxPi visuals, and supported a similar upregulated gene set enrichment profile following treatment. Tighter (eg, smaller in size) clusters were generally observed for rosiglitazone, acetaminophen, and d-galactosamine, with greater overlap between these 3 chemicals with each other and less overlap with HFPO-DA and GW7647 clusters as was also seen with the ToxPi visuals (Figure 6) and gene set activity concordance (Figure 4). PCA plots for the gene set aggregation results for B6129SF2/J mouse hepatocytes concurred with findings in CD-1 mouse hepatocytes (data provided in companion publication). As mentioned above, because of the lower number of significantly downregulated gene sets in rodent hepatocytes treated with HFPO-DA, GW7647, or rosiglitazone, aggregation analyses of downregulated gene sets were not able to be performed across chemical treatment groups and timepoints.

Overall, both concordance and gene set aggregation analyses demonstrated a consistent overlap in pathway-level responses between HFPO-DA and GW7647 in both mouse and rat hepatocytes. Gene sets related to fatty acid beta-oxidation, peroxisomes, and PPAR signaling were consistently among the most significantly enriched gene sets for both HFPO-DA and GW7647-treated hepatocytes. A lack of consistent and similar pathway-level responses were observed between HFPO-DA-treated rodent hepatocytes and those treated with rosiglitazone, acetaminophen, or d-galactosamine.

#### Upstream regulator predictions across chemical treatment groups in rodent hepatocytes

**Figure 7.**
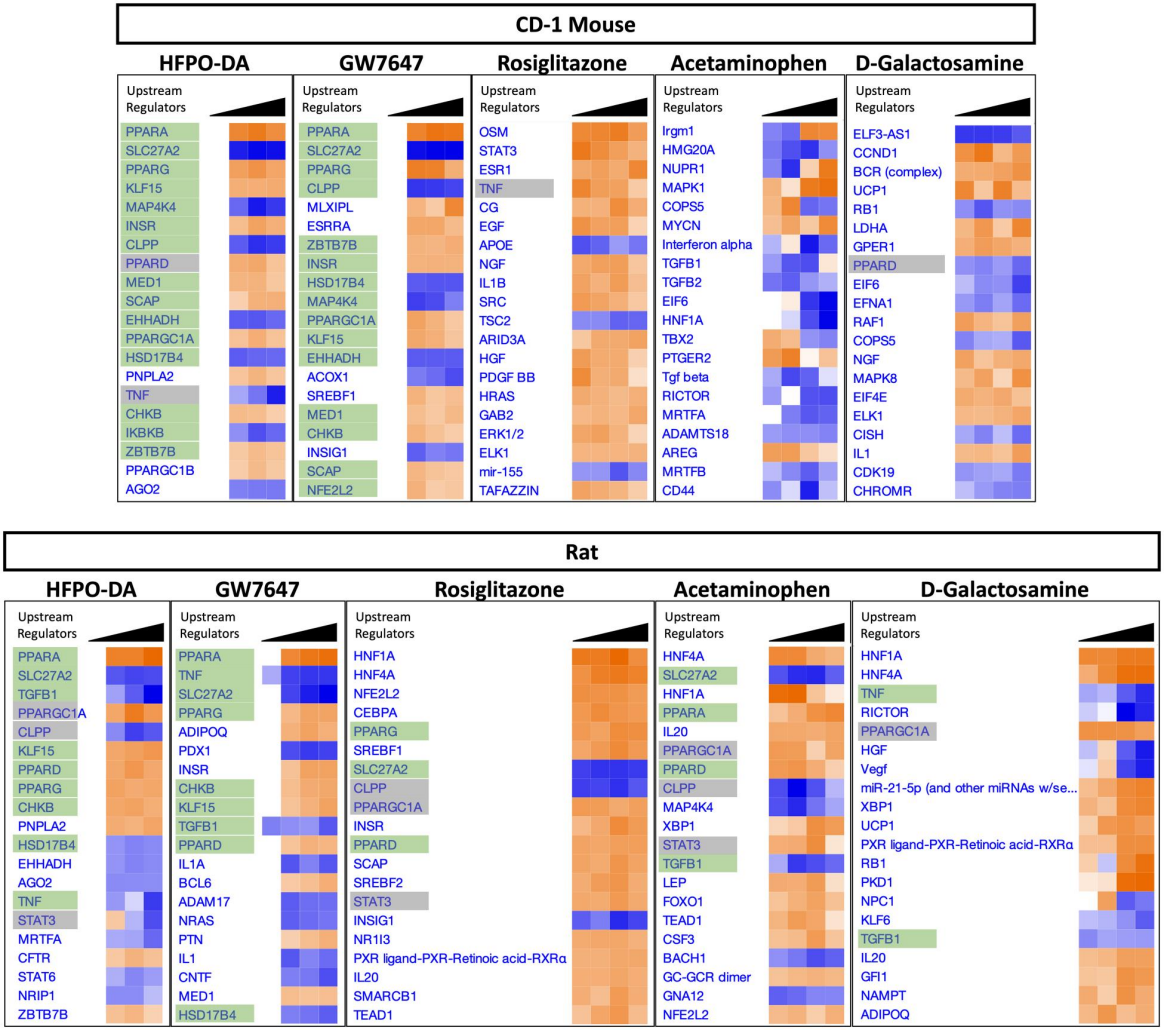
Top 20 predicted upstream regulators in mouse and rat hepatocytes at 24 h using IPA upstream analyses. Each column represents a different test concentration, with concentrations increasing from left to right for each chemical. Orange indicates predicted activation, and blue indicates predicted inhibition; the intensity of each color increases with the absolute *z*-score. Columns with no *z*-score prediction indicate chemical treatment groups with a low number DEGs and upstream regulator predictions were not able to be estimated. Predicted upstream regulators with green shading indicate upstream regulators that are predicted for both HFPO-DA and the positive control GW7647. Predicted upstream regulators with gray shading indicate upstream regulators that are in common between HFPO-DA and 1 or more of the other positive controls (ie, rosiglitazone, acetaminophen, d-galactosamine). Results for B6129SF2/J mouse hepatocytes available in companion publication.

The top 20 predicted upstream regulators identified using QIAGEN Ingenuity Pathway Analysis (IPA) for each timepoint in mouse and rat primary hepatocytes showed similar activation/inhibition patterns between HFPO-DA and GW7647 across timepoints (Figure 7 and Supplementary Figure 7). Conversely, patterns of predicted upstream regulator activation/inhibition for rosiglitazone, acetaminophen, and d-galactosamine were different, and generally inconsistent with predicted regulator activation/inhibition patterns for HFPO-DA and GW7647. PPARα was the top predicted upstream regulator in both CD-1 mouse and rat hepatocytes treated with HFPO-DA or GW7647 at 12 and 24 h. At 72 h, PPARα was still among the top predicted upstream regulators for HFPO-DA and GW7647 in mouse and rat hepatocytes; although, additional upstream regulators including PPARγ, PPARGCIA (peroxisome proliferator-activated receptor-gamma coactivator 1-alpha), HNF1α (hepatocyte nuclear factor 1-alpha) and SlC27A2 (very long-chain acyl-CoA synthetase) were among the top upstream regulators for both HFPO-DA and GW7647. Several of the genes underlying regulator predictions for PPARα are shared with PPARGCIA, HNF1α, SLC27A2, and PPARγ, as well as PPARδ, another PPAR subtype. As such, these transcription factors, enzymes, and nuclear receptors were also among the top predicted upstream regulators for both species. However, PPARγ-specific gene sets were generally not enriched in HFPO-DA or GW7647 exposed hepatocytes. In addition, as described above, PPARγ-specific gene sets were not enriched in rosiglitazone exposed hepatocytes and likely corroborate the low expression of PPARγ in the liver (Chawla *et al.*, 1994). Although gene sets specific to PPARδ are not currently available within the canonical pathway subcollection used for gene set enrichment analyses, pathways related to PPARδ are not expected to be enriched in hepatocytes as PPARδ is predominantly expressed in skeletal muscle (Holst *et al.*, 2003).

Overall, upstream regulator predictions in rodent hepatocytes complement findings from pathway-level analyses and predict a similar activation pattern of upstream regulators for HFPO-DA and GW7647-treated hepatocytes, with PPARα as the predominant top upstream regulator for both chemicals in mouse and rat hepatocytes.

#### Benchmark concentration modeling of gene expression data from rodent hepatocytes

**Figure 8.**
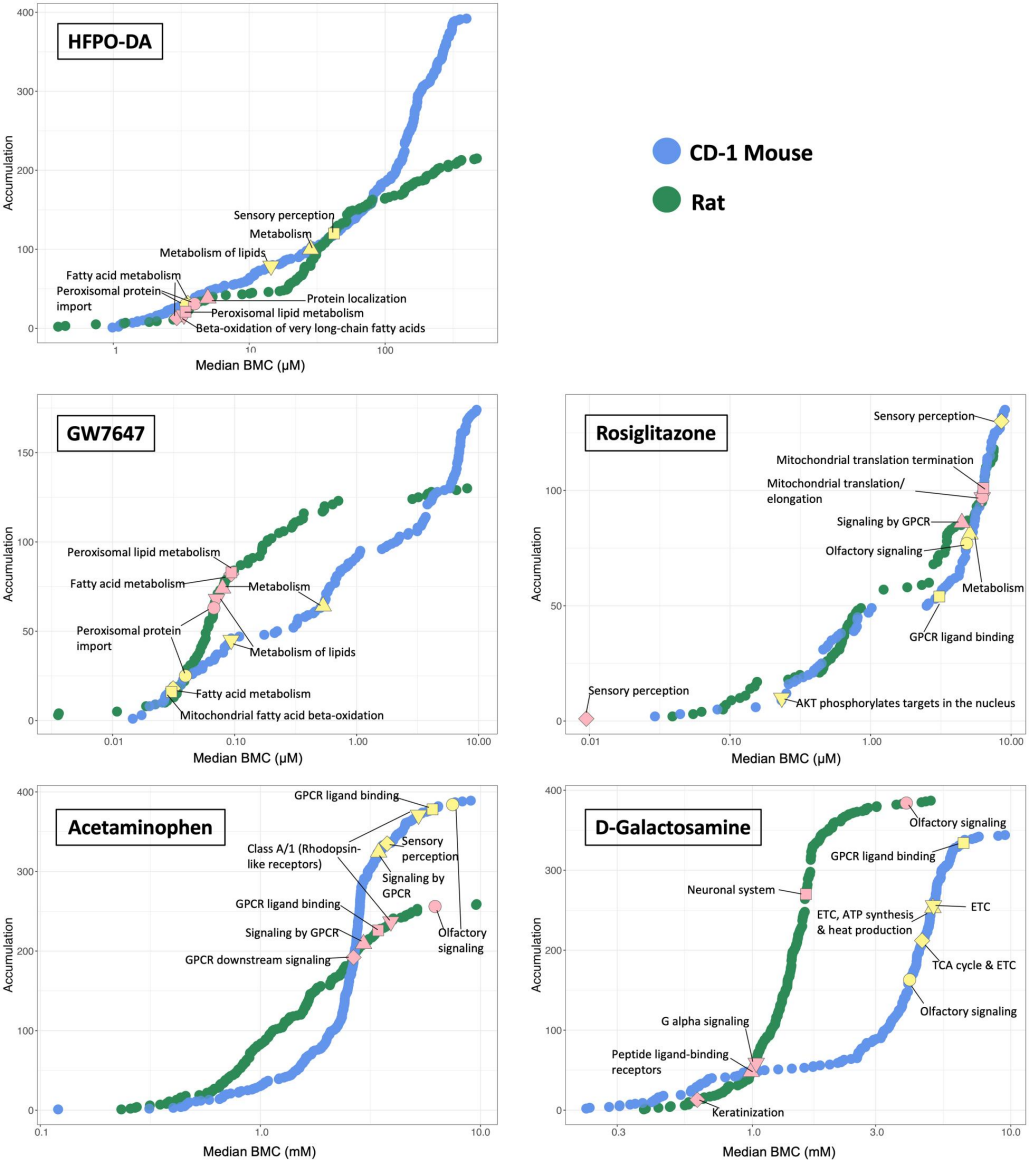
Accumulation plots of median BMCs for significantly enriched pathways (Fisher’s Exact 2-tail < 0.1) in mouse (blue points) and rat (green points) hepatocytes at 24 h. The top 5 most significantly enriched signaling pathways among concentration-responsive probes for each chemical are annotated by color-coded shapes. Yellow shapes indicate the top 5 most significantly enriched pathways for CD-1 mouse hepatocytes, and pink shapes indicate the top 5 most significantly enriched pathways for rat hepatocytes. Results for B6129SF2/J mouse hepatocytes available in companion publication. GPCR, G protein-coupled receptor; ETC, electron transport chain, TCA, tricarboxylic acid.

The concentration-response across all genes for each treatment group and species/strain was modeled using BMDExpress v2.3 (Phillips *et al.*, 2019) (Supplementary File 5). Results from the functional classification (ie, enrichment of signaling pathways) of significant concentration-responsive genes were similar to the hypergeometric gene set enrichment analyses described in the previous section (Supplementary File 6). Accumulation plots for the median BMC of significantly enriched signaling pathways are shown in Figure 8. The 5 most significantly enriched signaling pathways among concentration-responsive genes are also indicated in Figure 8. For example, the top-most significantly enriched signaling pathways in rodent hepatocytes treated with HFPO-DA or GW7647 for 24 h were related to fatty acid metabolism, peroxisomal lipid metabolism, peroxisomal protein import, and beta-oxidation of fatty acids. BMC medians of highly enriched signaling pathways specifically related to peroxisome activity or beta-oxidation occurred at the lower end of experimental concentration ranges (ie, below the second lowest concentration tested) for both HFPO-DA and GW7647, with median BMCs ranging from 2.93 to 3.96 and 0.03 to 0.094 µM, respectively in both mouse and rat hepatocytes (Figure 8), indicating that these signaling pathways are sensitive (ie, initial) transcriptomic responses in rodent hepatocytes following HFPO-DA and GW7647 treatment. Median BMCs for the top-most significantly enriched concentration-responsive pathways for rosiglitazone and the cytotoxic positive controls (acetaminophen and d-galactosamine) generally occurred between medium and high-test concentrations (Figure 8), suggesting that although these pathways had the greatest relative enrichment, they do not necessarily represent initial responses in rodent hepatocytes following treatment with these chemicals. Similar patterns for significantly enriched pathways were also noted at 12 and 72 h timepoints in rodent hepatocytes (see Supplementary File 6). Similar to the hypergeometric gene set enrichment analysis results for human hepatocytes, limited enrichment of gene sets was observed across chemicals and timepoints following functional classification of significant concentration-responsive genes in human hepatocytes, with significant concentration-responsive pathways generally limited to the cytotoxic positive controls at 24 and 72 h timepoints (Supplementary File 6).

Overall, these BMC analyses demonstrate broadly comparable responses in mouse and rat hepatocytes, with pathway enrichment indicating much lower BMC values (ie, more sensitive responses) for PPARα related pathways in HFPO-DA and GW7647-treated hepatocytes as compared with the other positive control chemicals evaluated.

#### Transcriptomic responses in human hepatocytes

As shown in Figure 3, the number of DEPs in human hepatocytes was generally more consistent across concentrations within a chemical treatment group compared with rodent hepatocytes, particularly at 24 h; in rodent hepatocytes the number of DEPs generally increased with increasing concentration with a chemical treatment group. In addition, unlike rodent hepatocytes, the DEPs in human hepatocytes resulted in few to no enriched gene sets or concentration-responsive pathways when treated with HFPO-DA, GW7647, or rosiglitazone, suggesting a low biological response at the pathway level in human hepatocytes following treatment with these compounds. In contrast to these PPARα/γ activating chemicals, pathway level responses were observed in human hepatocytes exposed to cytotoxic positive controls acetaminophen or d-galactosamine at 24 and 72 h (Supplementary Files 4 and 6). In addition, when a more relaxed gene set enrichment analysis (ie, GSEA method) was conducted (see Supplementary File 7 for methods) for human hepatocytes, upregulation of fatty acid metabolism and transport gene sets was observed at 24 h at the highest concentration tested for HFPO-DA-treated hepatocytes (Supplementary File 7). Gene sets related to PPAR signaling were upregulated at 72 h in GW7647-treated hepatocytes but did not meet adjusted *p* value threshold (*p* = .14). Similar gene sets related to innate immune responses were significantly downregulated in both HFPO-DA and GW7647-treated human hepatocytes. Gene sets associated with mitochondrial processes were among the most significantly enriched for rosiglitazone-treated hepatocytes using the relaxed gene set enrichment analysis.

**Figure 9.**
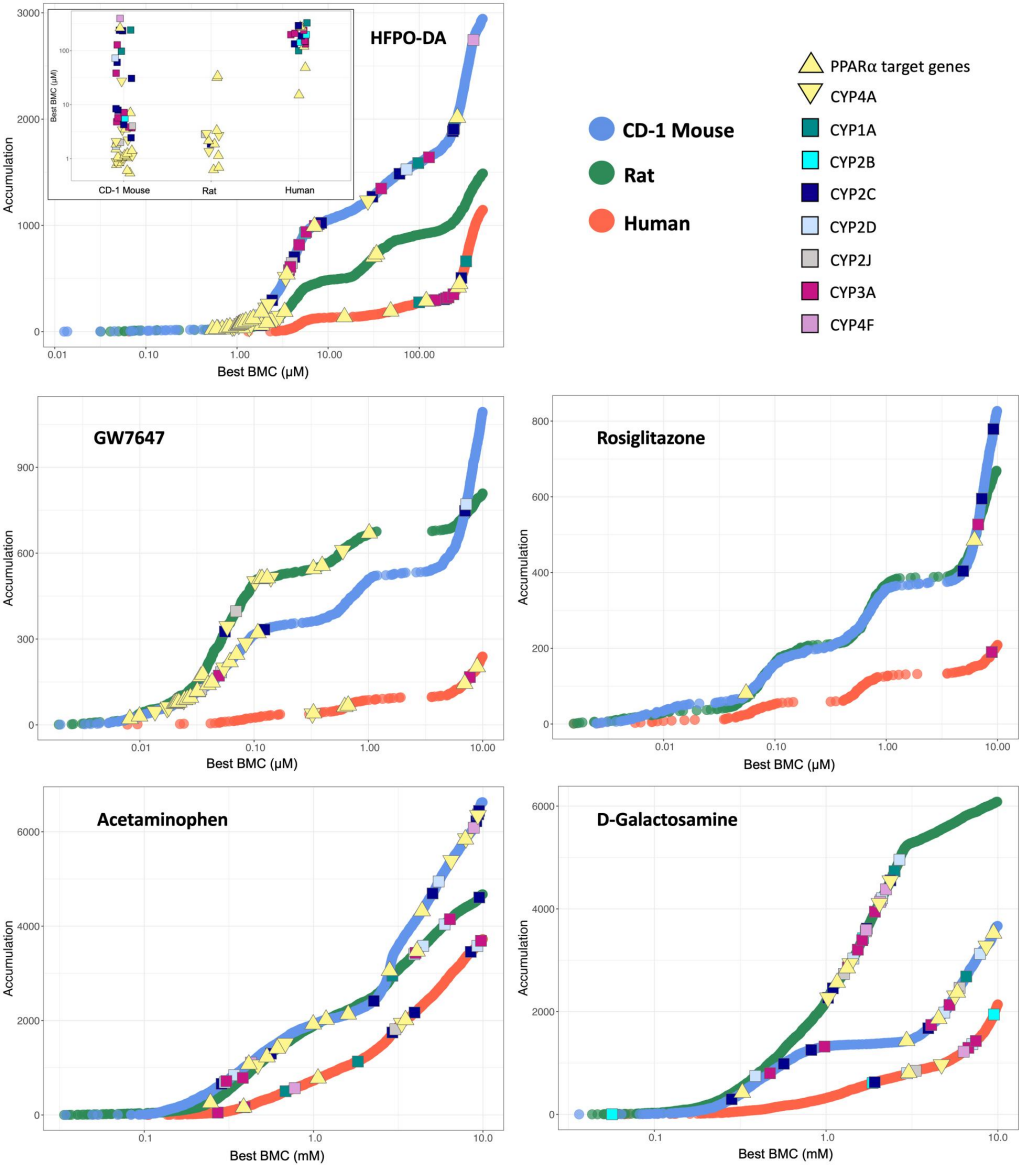
Accumulation plots of best BMCs among significant concentration-responsive probes (best fit *p* value ≥ .1) in rodent and human hepatocytes at 24 h. Mouse (CD-1), rat, and human concentration-responsive probes are indicated by blue, green, and orange points, respectively. Best BMCs of PPARα target genes and lipid metabolizing cytochrome P450’s (CYPs) are annotated by color-coded shapes. The PPARα target genes consist of 12 genes identified as HFPO-DA and/or GW7647-interacting genes highlighted in the ToxPi key in Figure 5. CYP4A is set apart from the other lipid metabolizing CYPs (denoted by a yellow downward-facing triangle shape) because it is the target CYP for PPARα. An inset plot for HFPO-DA is provided to show differences in best BMCs of PPARα target genes across hepatocyte species at low concentrations. Results for B6129SF2/J mouse hepatocytes available in companion publication.

Despite the lower pathway-level response in human hepatocytes compared with rodent hepatocytes exposed to HFPO-DA and PPARα/γ agonists, the concentration-response pattern of PPARα target genes, in addition to other known lipid metabolizing cytochrome P450 (CYP) genes, are generally similar across species for both HFPO-DA and GW7647-treated hepatocytes, with PPARα target genes consistently having lower BMCs than lipid metabolizing CYPs across timepoints (see Figure 9 for 24 h and Supplementary File 5 for other timepoints). However, this concentration-response pattern was not observed in hepatocytes treated with rosiglitazone, acetaminophen or d-galactosamine. These findings indicate that PPARα target genes have greater sensitivity to HFPO-DA and GW7647 treatment than other CYPs involved in lipid metabolism in both rodent and human hepatocytes. However, compared with BMCs for PPARα target genes in mouse and rat hepatocytes that were as low as 0.5 µM and 0.01 µM for HFPO-DA and GW7647, respectively, BMCs for PPARα target genes in human hepatocytes were more than a 10-fold higher for both HFPO-DA and GW7647, that is, 15.1 µM and 0.3 µM, respectively (see Figure 9 and inset graph for HFPO-DA in Figure 9). In addition, similar upstream regulators are predicted for HFPO-DA and GW7647, but not rosiglitazone, at higher concentrations in human hepatocytes across timepoints (Supplementary Figure 8).

Overall, the findings in human hepatocytes indicated limited pathway enrichment for nuclear receptor activators GW7647 and rosiglitazone as well as HFPO-DA. Among concentration-responsive genes in HFPO-DA and GW7647-treated human hepatocytes, results indicate PPARα-like activity at lower concentrations than other receptors responsible for CYP induction. However, compared with human hepatocytes, BMCs for PPARα target genes were more than 10-folder lower in rodent hepatocytes treated with HFPO-DA or GW7647, indicating human hepatocytes are less sensitive to activation of PPARα and PPARα target genes by these chemicals.

## Discussion

Whole transcriptomic analyses on primary rodent and human hepatocytes treated with HFPO-DA or positive controls demonstrate that transcriptomic responses in HFPO-DA-treated hepatocytes were consistent with responses in GW7647-treated hepatocytes for each species and strain evaluated. PPARα target genes and associated pathways were significantly upregulated in hepatocytes following treatment with HFPO-DA or the PPARα agonist, GW7647, at all 3 timepoints (12, 24, and 72 h). In addition, predictions for top upstream regulators were consistent between HFPO-DA and GW7647-treated hepatocytes for each species or strain. In contrast, a lack of concordance was observed at both the pathway and predicted regulator-level between hepatocytes treated with HFPO-DA and the PPARγ agonist (rosiglitazone) or cytotoxic agents (acetaminophen or d-galactosamine). These results provide further evidence for a PPARα-mediated MOA for liver effects in rodents exposed to HFPO-DA (Heintz *et al.*, 2023) and lack of evidence for PPARγ or cytotoxic MOAs that have been proposed for HFPO-DA (USEPA, 2021).

Across species, consistently greater transcriptomic responses were observed in rodent hepatocytes compared with pooled human hepatocytes at the pathway and predicted regulator-levels, especially for those treated with HFPO-DA or one of the other established nuclear receptor agonists (ie, GW7647 or rosiglitazone). Previous studies have also noted similar differences in gene and/or pathway-level responses between human and mouse (Rakhshandehroo *et al.*, 2009) or rat (Bjork *et al.*, 2011; McMullen *et al.*, 2020) hepatocytes treated with activators of PPARα or other hepatic nuclear receptors. Although the use of pooled human hepatocytes from 10 donors (5 male, 5 female) may also potentially contribute to a lower response, this approach is more representative of a population and included both Caucasian and Hispanic individuals ranging in age from 10 to 67 years (see Supplementary File 1). Concentration-response analyses showed that PPARα target genes in HFPO-DA or GW7647-treated hepatocytes consistently had lower BMCs than genes regulated by other nuclear receptors (eg, CAR or PXR) for all hepatocyte species evaluated—supporting activation of PPARα as the molecular initiating event for HFPO-DA and GW7647 in both rodent and human hepatocytes. However, as described previously, the downstream liver effects observed in rodents following PPARα activation (eg, proliferative responses and development of liver tumors) are not expected in humans as it is well-established that only the first KE of the PPARα MOA, PPARα activation, is shared across species (Corton *et al.*, 2018; Heintz *et al.*, 2023).

In addition to consistent transcriptomic responses between rat and mouse hepatocytes treated with HFPO-DA or GW7647, *in vitro* transcriptomic results presented herein for HFPO-DA-treated mouse hepatocytes complement *in vivo* hepatic transcriptomic results from mice orally exposed to HFPO-DA (Chappell *et al.*, 2020; Heintz *et al.*, 2022). However, between CD-1 and B6129SF2/J mouse hepatocyte strains, a slightly lower transcriptomic response in terms of the number of significant DEPs and enriched pathways was observed in B6129SF2/J hepatocytes treated with HFPO-DA, GW7647 or rosiglitazone. This slightly lower response in B6129SF2/J hepatocytes might be explained, in part, by the changes in hepatocyte morphology indicative of cell death observed at the 24 and 72 h timepoints for GW7674 and HFPO-DA (72 h only) in the high concentration treatment groups, although responses related to cytotoxicity were not observed at the transcriptomic level for either chemical nor was LDH release observed. Despite the slight difference in the overall level of response, the types of gene, pathway, and receptor-level responses were equivalent between mouse hepatocyte strains for each chemical tested.

*In vitro* transcriptomic results for the positive control chemicals used in the current study concurred with results previously reported in mechanistic studies for each chemical. For example, in addition to noting differences in transcriptomic response levels between human and rat hepatocytes treated with the PPARα agonist GW7647, McMullen *et al.* (2020) found similar gene ontology enrichment results in rat hepatocytes treated with GW7647 to what was observed in the current study for pathway enrichment results in both GW7674 and HFPO-DA-treated rat (and mouse) hepatocytes. These enriched pathways/gene ontology terms included the upregulation of lipid metabolism and beta-oxidation-related processes and downregulation of cell-cell communication and platelet activation/coagulation cascades. *In vitro* experiments with rosiglitazone are commonly conducted in adipocytes, the tissue with the highest PPARγ expression (Kersten, 2014). Although expressed at substantially lower levels in the liver, PPARγ regulates hepatic lipid uptake, triglyceride storage, and formation of lipid droplets (Wang *et al.*, 2020). In the current study, the most significantly enriched pathways in rodent hepatocytes treated with rosiglitazone were associated with regulation of cell function; however, pathways related to lipid particle composition, familial hyperlipidemia, and fatty acid metabolism were also enriched in rosiglitazone-treated mouse and rat hepatocytes.

As expected for chemicals known to elicit cytotoxicity, hepatocytes treated with acetaminophen or d-galactosamine generally had the greatest transcriptomic response based on number of DEPs and enriched pathways compared with the other chemicals tested. Mouse hepatocytes were previously reported to have greater susceptibility to acetaminophen-induced toxicity when compared with rat hepatocytes (Kučera *et al.*, 2017), this was also found in the current study as overt cytotoxicity was observed in mouse hepatocytes at earlier timepoints and lower concentrations compared with rat hepatocytes. Conversely, the opposite has been reported for d-galactosamine, with rats being more sensitive to d-galactosamine-induced hepatotoxicity and mice generally resistant until exposed to very high doses (Rahman and Hodgson, 2000). Cytotoxicity and pathway enrichment results corroborated these findings in the current study, as the top-most significantly enriched pathways (see Figure 7) and cytotoxicity occurred in rat hepatocytes at lower concentrations compared with mouse hepatocytes. Recently, a cytotoxicity gene set based on the expression of 10 genes has been developed from short-term rat toxicity studies (Glaab *et al.*, 2021). The expression of these 10 genes was examined across chemical treatment groups and timepoints in CD-1 mouse hepatocytes (Supplementary Figure 9). In general, these gene markers of cytotoxicity were more frequently perturbed in acetaminophen and d-galactosamine-treated hepatocytes, as well as hepatocytes treated with rosiglitazone.

Overall, transcriptomic responses in rodent and human hepatocytes treated with HFPO-DA demonstrate activation of PPARα based on increased expression of PPARα target genes and associated pathways. Pathway and receptor-level profiles for HFPO-DA-treated hepatocytes aligned closely with hepatocytes treated with an established PPARα agonist and had little to no overlap with other positive control chemicals for PPARγ activity or cytotoxicity. Compared with human hepatocytes, a greater transcriptomic response was consistently observed in rodent hepatocytes treated with HFPO-DA or the other established nuclear receptor agonists, indicating rodent hepatocytes are more sensitive to HFPO-DA treatment. Thus, consistent with previously published transcriptomic analyses, these results provide further evidence that the liver effects associated with HFPO-DA exposure in rodents are mediated through rodent-specific PPARα signaling mechanisms as part of the MOA for PPARα activator-induced rodent hepatocarcinogenesis. Based on the data herein, the liver effects observed *in vivo* for HFPO-DA are likely due solely to PPARα mediated mechanisms and not cytotoxicity or PPARγ mechanisms. Furthermore, the activation of PPARα (KE 1) was far weaker in human than rodent hepatocytes treated with HFPO-DA. Based on these findings and the larger body of evidence that PPARα activators do not elicit similar downstream KEs in humans, the liver effects in mice are not appropriate endpoints for use in the development of toxicity values for HFPO-DA in human health risk assessment. In addition, this novel *in vitro* study design can be applied to other PFAS compounds to help inform MOA and human relevance.

## Supplementary Material

kfae044_Supplementary_Data

## Data Availability

RNA sequencing data are publicly available at NCBI’s Gene Expression Omnibus (https://www.ncbi.nlm.nih.gov/geo/) (GEO series accession number GSE248251). All Supplementary Files (1–7) are available at DOI: 10.5061/dryad.stqjq2c94.

## References

[kfae044-B1] Anderson J. K. , Brecher R. W., Cousins I. T., DeWitt J., Fiedler H., Kannan K., Kirman C. R., Lipscomb J., Priestly B., Schoeny R., et al (2022). Grouping of PFAS for human health risk assessment: Findings from an independent panel of experts. Regul. Toxicol. Pharmacol. 134, 105226. 35817206 10.1016/j.yrtph.2022.105226

[kfae044-B2] Bjork J. A. , Butenhoff J. L., Wallace K. B. (2011). Multiplicity of nuclear receptor activation by PFOA and PFOS in primary human and rodent hepatocytes. Toxicology 288, 8–17. 21723365 10.1016/j.tox.2011.06.012

[kfae044-B3] Black M. B. , Stern A., Efremenko A., Mallick P., Moreau M., Hartman J. K., McMullen P. D. (2022). Biological system considerations for application of toxicogenomics in next-generation risk assessment and predictive toxicology. Toxicol. In Vitro 80, 105311. 35038564 10.1016/j.tiv.2022.105311

[kfae044-B4] Brown P. J. , Stuart L. W., Hurley K. P., Lewis M. C., Winegar D. A., Wilson J. G., Wilkison W. O., Ittoop O. R., Willson T. M. (2001). Identification of a subtype selective human PPARalpha agonist through parallel-array synthesis. Bioorg. Med. Chem. Lett. 11, 1225–1227. 11354382 10.1016/s0960-894x(01)00188-3

[kfae044-B5] Chappell G. A. , Thompson C. M., Wolf J. C., Cullen J. M., Klaunig J. E., Haws L. C. (2020). Assessment of the mode of action underlying the effects of GenX in mouse liver and implications for assessing human health risks. Toxicol. Pathol. 48, 494–508. 32138627 10.1177/0192623320905803 PMC7153225

[kfae044-B6] Chawla A. , Schwarz E. J., Dimaculangan D. D., Lazar M. A. (1994). Peroxisome proliferator-activated receptor (PPAR) gamma: Adipose-predominant expression and induction early in adipocyte differentiation. Endocrinology 135, 798–800. 8033830 10.1210/endo.135.2.8033830

[kfae044-B7] Corton J. C. (2023). PPARα activation leading to hepatocellular adenomas and carcinomas in rodents. Cancer AOP Workgroup. National Health and Environmental Effects Research Laboratory, Office of Research and Development, Integrated Systems Toxicology Division, US Environmental Protection Agency, Research Triangle Park, NC.

[kfae044-B8] Corton J. C. , Peters J. M., Klaunig J. E. (2018). The PPARalpha-dependent rodent liver tumor response is not relevant to humans: Addressing misconceptions. Arch. Toxicol. 92, 83–119. 29197930 10.1007/s00204-017-2094-7 PMC6092738

[kfae044-B9] Glaab W. E. , Holder D., He Y. D., Bailey W. J., Gerhold D. L., Beare C., Erdos Z., Lane P., Michna L., Muniappa N., et al (2021). Universal toxicity gene signatures for early identification of drug-induced tissue injuries in rats. Toxicol. Sci. 181, 148–159. 33837425 10.1093/toxsci/kfab038

[kfae044-B10] Heintz M. M. , Chappell G. A., Thompson C. M., Haws L. C. (2022). Evaluation of transcriptomic responses in livers of mice exposed to the short-chain PFAS compound HFPO-DA. Front. Toxicol. 4, 937168. 35832492 10.3389/ftox.2022.937168 PMC9271854

[kfae044-B11] Heintz M. M. , Haws L. C., Klaunig J. E., Cullen J. M., Thompson C. M. (2023). Assessment of the mode of action underlying development of liver lesions in mice following oral exposure to HFPO-DA and relevance to humans. Toxicol. Sci. 192, 15–29. 36629480 10.1093/toxsci/kfad004 PMC10025879

[kfae044-B1800] Heintz M. M. , Klaren W. D., East A. W., Haws L. C., McGreal S. R., Campbell R. R., Thompson C. M. (2024). Comparison of transcriptomic profiles between HFPO-DA and prototypical PPARa, PPARg, and cytotoxic agents in wild-type and PPARa knockout mouse hepatocytes. Toxicol. Sci. 200, 183–198. 10.1093/toxsci/kfae045 PMC11199908 38574385

[kfae044-B12] Holst D. , Luquet S., Nogueira V., Kristiansen K., Leverve X., Grimaldi P. A. (2003). Nutritional regulation and role of peroxisome proliferator-activated receptor delta in fatty acid catabolism in skeletal muscle. Biochim. Biophys. Acta 1633, 43–50. 12842194 10.1016/s1388-1981(03)00071-4

[kfae044-B13] Kersten S. (2014). Integrated physiology and systems biology of PPARα. Mol. Metab. 3, 354–371. 24944896 10.1016/j.molmet.2014.02.002 PMC4060217

[kfae044-B14] Kučera O. , Endlicher R., Rychtrmoc D., Lotková H., Sobotka O., Červinková Z. (2017). Acetaminophen toxicity in rat and mouse hepatocytes in vitro. Drug Chem. Toxicol. 40, 448–456. 27960556 10.1080/01480545.2016.1255953

[kfae044-B15] Lehmann J. M. , Moore L. B., Smith-Oliver T. A., Wilkison W. O., Willson T. M., Kliewer S. A. (1995). An antidiabetic thiazolidinedione is a high affinity ligand for peroxisome proliferator-activated receptor gamma (PPAR gamma). J. Biol. Chem. 270, 12953–12956. 7768881 10.1074/jbc.270.22.12953

[kfae044-B16] Love M. I. , Huber W., Anders S. (2014). Moderated estimation of fold change and dispersion for RNA-seq data with DESeq2. Genome Biol. 15, 550. 25516281 10.1186/s13059-014-0550-8 PMC4302049

[kfae044-B17] McMullen P. D. , Bhattacharya S., Woods C. G., Pendse S. N., McBride M. T., Soldatow V. Y., Deisenroth C., LeCluyse E. L., Clewell R. A., Andersen M. E. (2020). Identifying qualitative differences in PPARα signaling networks in human and rat hepatocytes and their significance for next generation chemical risk assessment methods. Toxicol. In Vitro 64, 104463. 31628012 10.1016/j.tiv.2019.02.017

[kfae044-B18] Mudra D. R. , Parkinson A. (2001). Preparation of hepatocytes. Curr. Protoc. Toxicol. **Chapter 14**, Unit 14.2. 11-14.12.13. 10.1002/0471140856.tx1402s08 23045038

[kfae044-B19] NTP. (2018). NTP research report on National Toxicology Program approach to genomic dose-response modeling. Research report 5. National Toxicology Program. U.S. Department of Health and Human Services, Research Triangle Park, NC. 30321009

[kfae044-B20] Phillips J. R. , Svoboda D. L., Tandon A., Patel S., Sedykh A., Mav D., Kuo B., Yauk C. L., Yang L., Thomas R. S., et al (2019). BMDExpress 2: Enhanced transcriptomic dose-response analysis workflow. Bioinformatics 35, 1780–1782. 30329029 10.1093/bioinformatics/bty878 PMC6513160

[kfae044-B21] Rahman T. M. , Hodgson H. J. (2000). Animal models of acute hepatic failure. Int. J. Exp. Pathol. 81, 145–157. 10762442 10.1046/j.1365-2613.2000.00144.x PMC2517718

[kfae044-B22] Rakhshandehroo M. , Hooiveld G., Müller M., Kersten S. (2009). Comparative analysis of gene regulation by the transcription factor PPARalpha between mouse and human. PLoS One 4, e6796. 19710929 10.1371/journal.pone.0006796 PMC2729378

[kfae044-B23] Shah I. , Bundy J., Chambers B., Everett L. J., Haggard D., Harrill J., Judson R. S., Nyffeler J., Patlewicz G. (2022). Navigating transcriptomic connectivity mapping workflows to link chemicals with bioactivities. Chem. Res. Toxicol. 35, 1929–1949. 36301716 10.1021/acs.chemrestox.2c00245 PMC10483698

[kfae044-B08643112] Subramanian A. , Tamayo P., Mootha V. K., Mukherjee S., Ebert B. L., Gillette M. A., Paulovich A., Pomeroy S. L., Golub T. R., Lander E. S., et al (2005). Gene set enrichment analysis: A knowledge-based approach for interpreting genome-wide expression profiles. Proc. Natl. Acad. Sci. U S A. 102, 15545–15550. 16199517 10.1073/pnas.0506580102 PMC1239896

[kfae044-B24] Thompson C. M. , Fitch S. E., Ring C., Rish W., Cullen J. M., Haws L. C. (2019). Development of an oral reference dose for the perfluorinated compound GenX. J. Appl. Toxicol. 39, 1267–1282. 31215065 10.1002/jat.3812 PMC6771874

[kfae044-B25] Thompson C. M. , Heintz M. M., Wolf J. C., Cheru R., Haws L. C., Cullen J. M. (2023). Assessment of mouse liver histopathology following exposure to HFPO-DA with emphasis on understanding mechanisms of hepatocellular death. Toxicol. Pathol. 51, 4–14. 36987989 10.1177/01926233231159078 PMC10278389

[kfae044-B26] USEPA. (2021). Human health toxicity values for hexafluoropropylene oxide (HFPO) dimer acid and its ammonium salt (CASRN 13252-13-6 and CASRN 62037-80-3) also known as “GenX chemicals.” EPA document number: 822r-21-010. U.S. Environmental Protection Agency Office of Water (4304t) Health and Ecological Criteria Division, Washington, DC.

[kfae044-B27] Varemo L. , Nielsen J., Nookaew I. (2013). Enriching the gene set analysis of genome-wide data by incorporating directionality of gene expression and combining statistical hypotheses and methods. Nucleic Acids Res. 41, 4378–4391. 23444143 10.1093/nar/gkt111 PMC3632109

[kfae044-B28] Wang Y. , Nakajima T., Gonzalez F. J., Tanaka N. (2020). PPARs as metabolic regulators in the liver: Lessons from liver-specific PPAR-null mice. Int. J. Mol. Sci. 21, 2061. 32192216 10.3390/ijms21062061 PMC7139552

[kfae044-B29] Yeakley J. M. , Shepard P. J., Goyena D. E., VanSteenhouse H. C., McComb J. D., Seligmann B. E. (2017). A trichostatin a expression signature identified by TempO-Seq targeted whole transcriptome profiling. PLoS One 12, e0178302. 28542535 10.1371/journal.pone.0178302 PMC5444820

