# Supplementary material for: Comparison of transcriptomic profiles between HFPO-DA and prototypical PPARα, PPARγ, and cytotoxic agents in mouse, rat, and pooled human hepatocytes
Source: Toxicol Sci. 2024 Apr 4;200(1):165–82. doi: 10.1093/toxsci/kfae044 (PMC11199992; doi:10.1093/toxsci/kfae044)
Supplement: kfae044_Supplementary_Data [file kfae044_supplementary_data.docx]

**Supplementary Figures & Tables**

**Figure S1. LDH release measured at 12, 24 and 72 h in control and treated CD-1 mouse, B6129SF2/J mouse, SD rat, or human hepatocytes.**

**Figure S2. Principal component analysis plot of SD rat (A), B6129SF2/J mouse (B), and human (C) hepatocyte samples.** The chemical treatment group of each sample is indicated by color-coded shapes, with shape and color indicating chemical treatment and size indicating concentration level (see legend).

**Figure S3. Principal component analysis and scree plots of CD-1 mouse (A), B6129SF2/J mouse (B), SD rat (C), and human (D) hepatocyte samples by timepoint.** The chemical treatment group of each sample is indicated by color-coded shapes, with shape and color indicating chemical treatment and size indicating concentration level (see legend).

**Figure S4. Hierarchical cluster analyses of** **CD-1 mouse (A), B6129SF2/J mouse (B), SD rat (C), and human (D) hepatocyte samples by species/strain as well as all samples together (E).** Unsupervised clustering according to Euclidean distances was demonstrated by the ordering of individual hepatocyte samples, with distances depicted by dendrograms. Color and grayscale identifiers are assigned to each sample across the bottom of the hierarchical clustering figure according to species/strain (yellow, turquoise, pink and purple for CD-1 mouse, B6129SF2/J mouse, SD rat and human, respectively), timepoint (grayscale increasing in darkness with increasing exposure duration), chemical treatment (light gray for solvent controls, green, blue, red, orange and purple for HFPO-DA, GW7674, rosiglitazone, acetaminophen and d-galactosamine, respectively), and concentration (grayscale increasing in darkness with increasing concentration). Hierarchical clustering across hepatocyte samples was performed using the following methods. Mouse or rat probes were converted to human gene names using the R package biomaRt (v2.56.1) based on the Ensembl genome database. For genes for which multiple probes were used to measure expression, the probe with the highest mean sequencing count across all samples was used in hierarchical cluster analysis. Normalized gene expression data for each species/strain were collated, and whole transcriptome data were subset using the National Toxicology Program’s S1500+ gene set list for humans (Mav et al. 2018). Samples were clustered using normalized expression level per sample per probe, which was estimated by the distance of each individual sample from the mean expression level for that probe across all samples. R packages dendextend (v1.17.1), stringr (v1.5.1) and stats (base version) were used to conduct the analyses in R (v4.3.1).

**Figure S5. Number of significant (FDR < 10%) upregulated (top row) and downregulated (bottom row) DEPs (relative to controls) in primary hepatocytes for each species/strain and chemical tested at 12 h (A) and 72 h (B).** Each row represents a different chemical and each column represents a different test concentration, with concentrations increasing from left to right. An “*” indicates that cytotoxicity was observed at this concentration and timepoint.

**Figure S6. Upregulated gene set activity concurrence and concordance analyses**. Comparison of gene set activity concurrence and concordance results across CD-1, B6129SF2/J and rat hepatocytes at the second-highest concentration tested for each chemical at 12 h (A) or 72 h (B) or comparing cytotoxic positive controls (acetaminophen and d-galactosamine) at the second-lowest concentration to HFPO-DA and other positive controls (GW7647 and rosiglitazone) at the second-highest concentration tested at 12 h (C), 24 h (D), or 72 h (E). Activity concurrence matrices show the number of instances when there is agreement and disagreement in the active/inactive (i.e., significant/not significant) upregulated gene sets, comparing HFPO-DA to each positive control. The Jaccard index method was then applied to activity concurrence results to estimate relative concordance of active gene sets between HFPO-DA and each positive control. Greater color intensity and higher scores for concordance plots indicate greater overlap between active gene sets for two chemicals.

**Figure S7. Top 20 predicted upstream regulators in mouse and rat hepatocytes at 12 h (A) and 72 h (B) using IPA upstream analyses.** Each column represents a different test concentration, with concentrations increasing from left to right for each chemical. Orange indicates predicted activation, and blue indicates predicted inhibition; the intensity of each color increases with the absolute z-score. Columns with no z-score prediction indicate chemical treatment groups with a low number DEGs and upstream regulator predictions were not able to be estimated.

**Supplementary Figure S8. Top 20 predicted upstream regulators across rodent and human hepatocytes across timepoints using IPA upstream analyses.** Predicted upstream regulators at 12 h (A), 24 h (B), and 72 h (C). Each column represents a different test concentration, with concentrations increasing from left to right for each chemical. Orange indicates predicted activation, and blue indicates predicted inhibition; the intensity of each color increases with the absolute z-score.

**Supplementary Figure S9. Heatmap of significant differentially expressed genes associated with cytotoxicity in CD-1 mouse hepatocytes.** Cytotoxicity gene set described in Corton et al. (2020) and Glaab et al. (2021). Rosi = rosiglitazone, Ace = acetaminophen, Dgal = d-galactosamine

**Table S1. Number of samples remaining in each treatment group following removal of samples that failed sequencing quality criteria (see Supplementary File S1 Table 3).**

* Treatment group was removed from transcriptomic analyses.
